# Automatic triage of twelve-lead electrocardiograms using deep convolutional neural networks: a first implementation study

**DOI:** 10.1093/ehjdh/ztad070

**Published:** 2023-11-08

**Authors:** Rutger R van de Leur, Meike T G M van Sleuwen, Peter-Paul M Zwetsloot, Pim van der Harst, Pieter A Doevendans, Rutger J Hassink, René van Es

**Affiliations:** Department of Cardiology, University Medical Center Utrecht, Heidelberglaan 100, Utrecht 3584 CX, The Netherlands; Department of Cardiology, University Medical Center Utrecht, Heidelberglaan 100, Utrecht 3584 CX, The Netherlands; Department of Cardiology, University Medical Center Utrecht, Heidelberglaan 100, Utrecht 3584 CX, The Netherlands; Department of Cardiology, University Medical Center Utrecht, Heidelberglaan 100, Utrecht 3584 CX, The Netherlands; Department of Cardiology, University Medical Center Utrecht, Heidelberglaan 100, Utrecht 3584 CX, The Netherlands; Netherlands Heart Institute, Utrecht, The Netherlands; Central Military Hospital, Utrecht, The Netherlands; Department of Cardiology, University Medical Center Utrecht, Heidelberglaan 100, Utrecht 3584 CX, The Netherlands; Department of Cardiology, University Medical Center Utrecht, Heidelberglaan 100, Utrecht 3584 CX, The Netherlands

**Keywords:** Electrocardiography, Triage, Deep learning, Implementation

## Abstract

**Aims:**

Expert knowledge to correctly interpret electrocardiograms (ECGs) is not always readily available. An artificial intelligence (AI)-based triage algorithm (DELTAnet), able to support physicians in ECG prioritization, could help reduce current logistic burden of overreading ECGs and improve time to treatment for acute and life-threatening disorders. However, the effect of clinical implementation of such AI algorithms is rarely investigated.

**Methods and results:**

Adult patients at non-cardiology departments who underwent ECG testing as a part of routine clinical care were included in this prospective cohort study. DELTAnet was used to classify 12-lead ECGs into one of the following triage classes: normal, abnormal not acute, subacute, and acute. Performance was compared with triage classes based on the final clinical diagnosis. Moreover, the associations between predicted classes and clinical outcomes were investigated. A total of 1061 patients and ECGs were included. Performance was good with a mean concordance statistic of 0.96 (95% confidence interval 0.95–0.97) when comparing DELTAnet with the clinical triage classes. Moreover, zero ECGs that required a change in policy or referral to the cardiologist were missed and there was a limited number of cases predicted as acute that did not require follow-up (2.6%).

**Conclusion:**

This study is the first to prospectively investigate the impact of clinical implementation of an ECG-based AI triage algorithm. It shows that DELTAnet is efficacious and safe to be used in clinical practice for triage of 12-lead ECGs in non-cardiology hospital departments.

## Graphical Abstract

**Graphical Abstract:**
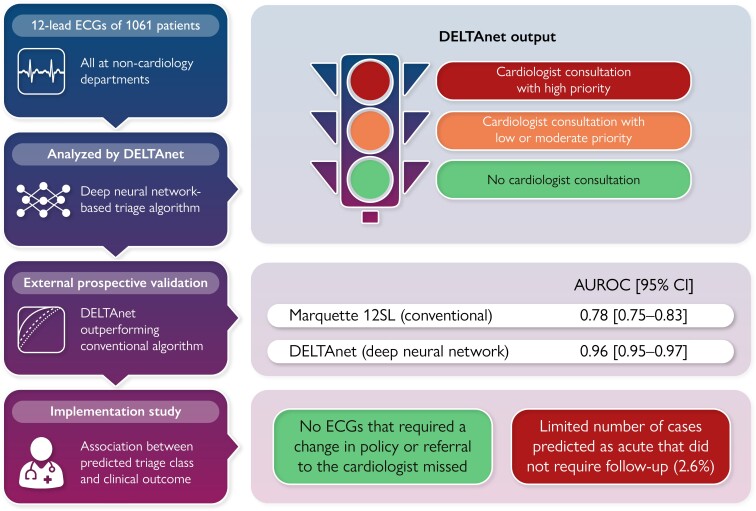
Overview of the study and its outcomes. AUROC, area under the receiver operating curve; CI, confidence interval; ECG, electrocardiogram.

## Introduction

Correct and timely interpretation of the electrocardiogram (ECG) is important for accurate diagnosis of a variety of cardiac abnormalities, as early treatment results in lower mortality and decreases disease burden for life-threatening cardiac disorders.^1–3^ Expert knowledge to interpret ECGs is often not readily available, especially in pre-hospital care and non-cardiology departments.^4–6^ Accurately prioritizing which ECGs need expert attention could lead to improvements in time to treatment and enhance the cost-effectiveness of current healthcare.^7,8^

Recent advancements in the field of artificial intelligence (AI) have shown that deep neural networks (DNNs) can learn to interpret ECGs with high accuracy.^9^ Previous studies have shown that DNNs can be used to detect many separate ECG abnormalities, such as different rhythm and conduction disorders and myocardial ischaemia.^10–14^ Deep neural networks have also been used to improve triage of ECGs by predicting 7-day mortality using the ECG only.^15^ One study showed that implementing an AI-based ST-elevation myocardial infarction algorithm successfully reduces door-to-balloon times in pre-hospital care.^16^

Our group developed a comprehensive DNN-based triage algorithm (DELTAnet) that is able to consistently triage all 12-lead hospital ECGs.^17^ DELTAnet was trained to classify each ECG into one of the following four classes based on how quickly a cardiologist should be consulted: (i) normal, no action needed; (ii) abnormal not acute, consultation with low priority; (iii) subacute, consultation with moderate priority; or (iv) acute, consultation with high priority. This algorithm was validated in an expert-annotated test set and shows potential to support physicians in comprehensive triage and decision-making regarding the prioritization of a newly acquired ECG.

Despite the rise in AI-optimized ECG interpretation approaches, clinical implementation of these algorithms is limited. Essential steps should be completed before clinical implementation is possible: (i) development and internal validation, (ii) external validation in other populations, and (iii) assessment of the implementation of the model in clinical practice with its impact on patient outcomes.^18^ Most studies regarding automated ECG applications address the first two phases, but their implications for implementation remain unclear. In this study, we aim to prospectively validate the performance of DELTAnet and investigate the impact of possible implementation of DELTAnet in clinical practice when applied to 12-lead ECGs from multiple non-cardiology hospital departments.

## Methods

### Study setting and participants

We conducted a prospective, single-centre, consecutive, and observational cohort study with adult inpatients who underwent ECG testing as a part of routine clinical care at University Medical Center Utrecht (UMCU, Utrecht, the Netherlands). Patients were included when their ECG was acquired in one of the following departments: the emergency room (ER), pre-operative screening department (POS), a non-cardiology ward, or a non-cardiology outpatient clinic between 1 and 31 October 2019. All ECGs were interpreted by a cardiologist or cardiology resident as part of the regular clinical workflow. Patients were excluded if their ECG was of insufficient quality, as annotated by the overreading physician. For patients with multiple ECGs acquired during their hospital stay, only the first ECG was selected for analysis. All ECGs were acquired using a General Electric MAC 5500 (GE Healthcare, Chicago, IL, USA), and electrodes could have been placed both in the standard or Mason–Likar configuration. The study was conducted under a protocol approved by the UMCU Institutional Review Board using a waiver of written informed consent.

### Triage classification of the ECG

We used a previously described deep learning–based triage algorithm (DELTAnet) that was developed and validated for comprehensive triage of 12-lead ECGs.^17^ In short, DELTAnet is a 37-layer convolutional neural network trained to triage ECG using a data set of 336,835 ECGs from 142,040 patients. For training, the physician annotations of each ECG were translated into one of the triage classes based on predefined criteria, and these triage classes were used to train the algorithm. Hyperparameters were tuned using a combination of manual tuning and random grid search on a subset of 5% of the training data set. DELTAnet was validated on an export-annotated test set of 984 ECGs from 984 patients. The algorithms output one of four triage categories, based on how quickly a cardiologist has to be consulted: (i) normal, (ii) not acute abnormal (consultation with low priority), (iii) subacute abnormal (consultation with moderate priority), and (iv) acute abnormal (consultation with high priority). For this study, custom software automatically extracts the raw ECG data from the MUSE system (GE Healthcare, Chicago, IL, USA) and this data was then triaged by the DELTAnet algorithm on a standard desktop computer. The DELTAnet prediction was not shown to the physician in this study.

**Figure 1:**
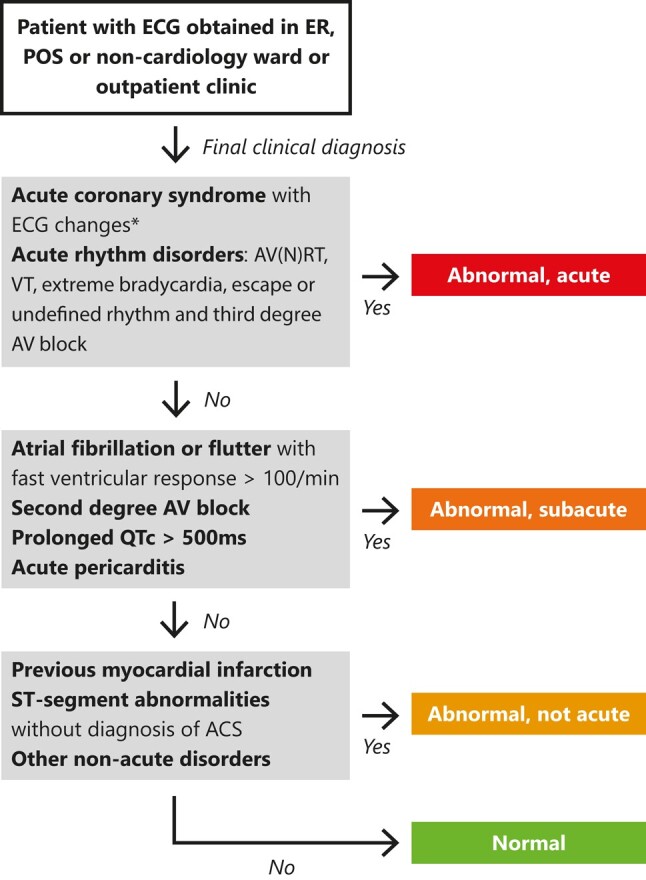
Labelling into triage classes as based on final clinical diagnosis. For few cases where the cardiologist-annotated diagnosis was not clear (e.g. whether specific or non-specific ST abnormalities), the ECG was assessed to determine the appropriate category. When multiple diagnoses were visible on the ECG, the highest triage class was chosen. All other non-acute disorders can be found in Supplementary material online, *Figure S1*. *ECG changes were defined as ST-segment deviations or T-wave changes associated with ischaemia. ACS, acute coronary syndrome; AV(N)RT, atrioventricular (nodal) re-entry tachycardia; VT, ventricular tachycardia; AV block, atrioventricular block; AF, atrial fibrillation.

We evaluated its performance by comparing the predicted triage classes with the triage classes as based on the final clinical diagnosis. In the development study, the model was trained and validated using only the physician annotation of the ECG categorized into one of the triage categories. Detailed ECG interpretation also needs additional clinical information, such as patient history, previous ECGs, and results of other tests. In the current analysis, the final clinical diagnosis was therefore extracted from medical record data and used to determine the clinical triage classes using the flowchart in *Figure 1* and the diagnostic statement to triage class matrix in Supplementary material online, *Figure S1*. The major difference between the current class definition and the one used for training is that ST-segment abnormalities are classified as either acute or not acute based on the outcomes of laboratory tests and coronary angiography.

For comparison purposes, we sought a commercially available and widely used conventional rule-based (i.e. not deep learning–based) algorithm for interpretation of 12-lead ECGs. The Marquette 12SL algorithm (GE Healthcare, Chicago, IL, USA) was selected, as it is used in all GE ECG systems and currently provides the computerized interpretation of the ECG in our hospital.^19^ Marquette 12SL diagnostic statements were manually mapped to triage classes based on Supplementary material online, *Figure S1*.

### Association with clinical care and outcomes

Currently, in our hospital, all ECGs acquired at non-cardiology departments are systematically overread by a cardiologist or cardiology resident within 24 h or faster when another physician asks for a consult. This a time-consuming task, which places a heavy logistic burden on clinical practice. To optimize this process, DELTAnet recommends the physician whether cardiologist consultation or overreading of the ECG is necessary and within which timeframe. Normal ECGs are no longer overread, while for acute ECGs, consultation is immediate. To assess the effect of implementing the DELTAnet recommendation in clinical practice, the association between the predicted triage class and the currently chosen management for this patient was evaluated. For all patients, the following events were logged: cardiologist consultation, whether there was a change in patient management (diagnostics, a medication change, or a cardiac procedure, such as electrical cardioversion or coronary angiography), follow-up appointment at a cardiology clinic, the final clinical diagnosis (whether cardiac/non-cardiac), and clinical outcomes (length of hospital stay and in-hospital mortality). We evaluated whether using DELTAnet to guide physicians resulted in similar management for patients as in current clinical practice in our hospital.

### Outcome measures

The outcome of this study is the expected impact of future implementation of DELTAnet into clinical practice in non-cardiology wards. We evaluated the classification performance as compared with the final clinical diagnosis in both the overall cohort and in several subgroups (age, gender, hospital location). Moreover, we compared the management of the patients between the predicted triage classes and focus on two outcomes: (i) no important undertriage, defined as ECGs as normal that should have required cardiac follow-up, and (ii) a limited proportion of overtriage, defined as ECGs predicted as acute that did not require any cardiac follow-up or had no final diagnosis of cardiac disease.

### Statistical analyses

For descriptive analysis, proportions and percentages and means with standard deviations (SD), or medians with interquartile ranges (IQR) when data were not normally distributed, were calculated. Overall classification performance was evaluated in terms of the unweighted mean of all pairwise concordance-statistics [c-statistics, equivalent to area under the receiver operating curve (AUROC)].^20,21^ This method is robust to class imbalance and calculates the AUROC for all pairs of classes. Given the number of classes *c*, any pair of classes *i* and *j*, and the measure of separability between two classes $\hat{A}$, this metric is defined as follows:^21^


$$M=\;\frac{2}{c(c-1)}\underset{i<j}{\sum }\;\hat{A}(i,\;j)$$

For category-specific performance, we assessed c-statistics, sensitivity, specificity, positive predictive values (PPV), and negative predictive values (NPV). All category-specific measures were applied in a one-vs.-other approach. To estimate the 95% confidence interval (CI) of the performance metrics, we used 2000 rounds of bootstrapping. C-statistics were compared using permutation tests. The TRIPOD guidelines were followed where applicable.^22^

## Results

### Patient characteristics

A total of 1061 patients were found eligible, and 48 were excluded due to technically insufficient recording quality of the ECG. The distribution of predicted triage categories was unbalanced with the most recordings being normal (52%) and the least belonging to the subacute group (4%). Most ECGs were acquired at the ER (42%), and the smallest subset contained ECGs obtained at non-cardiology wards (14%). *Table 1* summarizes the patient characteristics, hospital locations, and predicted triage class distributions of the data set.

**Table 1:** Patient characteristics and distributions in hospital location and triage classes

	Overall
	*n* = 1013
Demographics	
Age, median (IQR)	64 (52–73)
Female sex, *n* (%)	439 (43%)
BMI, median (IQR)	29 (25–30)
Medical history	
History of cardiovascular disease, *n* (%)	506 (49%)
Cardiac procedure in history, *n* (%)	275 (27%)
Risk factors	
Hypertension	467 (45%)
Diabetes	298 (29%)
High cholesterol	232 (23%)
Smoking	464 (46%)
Location, *n* (%)	
Emergency room	430 (42%)
Non-cardiology outpatient clinic	253 (25%)
Non-cardiology ward	143 (14%)
Pre-operative screening	187 (18%)
Predicted triage class, *n* (%)	
Normal	529 (52%)
Abnormal, not acute	373 (37%)
Abnormal, subacute	29 (3%)
Abnormal, acute	82 (8%)

BMI, body mass index; IQR, interquartile range.

### Classification performance

**Figure 2:**
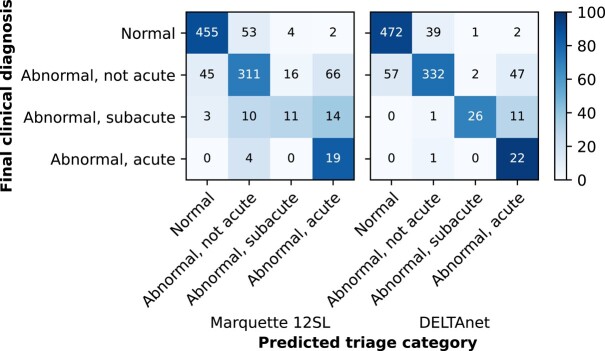
Confusion matrix comparing Marquette 12SL and DELTAnet predictions to the clinical triage classes (based on final clinical diagnosis).

The overall classification performance of DELTAnet, as measured by the unweighted mean of pairwise c-statistics, was 0.96 (95% CI 0.95–0.97) when comparing the predicted triage classes with the final clinical diagnosis. DELTAnet outperformed the Marquette 12SL algorithm, which had an unweighted mean of pairwise c-statistics of 0.78 (95% CI 0.75–0.83, *P* < 0.001). The c-statistics, sensitivities, specificities, positive predictive values, and negative predictive values per triage category of DELTAnet are shown in *Table 2* and the corresponding confusion matrix in *Figure 2*. Classification performance was good for all subgroups (see Supplementary material online, *Figure S2*). None of the pairwise combinations showed significant differences between subgroups.

**Table 2:** Performance measures per triage class comparing predicted triage classes by DELTAnet with clinical triage classes

	Normal	Abnormal not acute	Abnormal subacute	Abnormal acute
c-statistic (95% CI)	0.95 (0.94–0.96)	0.91 (0.89–0.93)	0.99 (0.98–1.00)	0.98 (0.97–0.99)
Sensitivity	0.92	0.76	0.68	0.96
Specificity	0.89	0.93	1.0	0.94
PPV	0.89	0.89	0.90	0.27
NPV	0.91	0.83	0.99	1.0

C-statistics (concordance statistic), sensitivities, specificities, and positive and negative predictive values are all calculated in a one-vs.-other approach.

CI, confidence interval; PPV, positive predictive value; NPV, negative predictive value.

### Under- and overtriage

For 59 patients (5.6%), DELTAnet predicted a lower triage class than was determined by the final clinical diagnosis (undertriage). Most undertriage consisted of patients classified as not acute but predicted to be normal by DELTAnet (57/59, 97% of all undertriage). These patients were classified as not acute based on non-specific ST abnormalities (21/57, 37%), incomplete right bundle branch block (9/57, 16%), aspecific intraventricular conduction delay (7/57, 12%), previous ischaemia (6/57, 11%), bradycardia <50 b.p.m. (6/57, 11%), left ventricular hypertrophy (4/57, 7%), low QRS voltage (2/57, 4%), or a combination of mentioned abnormalities (2/57, 4%). One undertriage case (1/59, 2%) concerned a patient classified as acute but predicted to be non-acute by DELTAnet. This represented a patient that presented at the ER with chest pain, atypical ST-elevation in leads V2 and V3 without reciprocal depression, and low troponin. The patient was clinically triaged as acute because the final diagnosis was unstable angina with coronary stenosis on coronary angiography (see Supplementary material online, *Figure S3*). The last undertriage case (1/59, 2%) reflected a patient classified as subacute because of QTc > 500 ms but was predicted as non-acute by DELTAnet.

For 102 patients (9.6%), DELTAnet predicted a higher triage class than was determined by the final clinical diagnosis (overtriage). Most overtriage occurred for patients classified as non-acute but predicted to be acute by DELTAnet (47/102, 46%). Overpredictions mainly represented patients with ST abnormalities on the ECG but no final clinical diagnosis of acute ischaemia (39/47, 72%) (see Supplementary material online, *Figure S4*). Other non-acute overpredictions concerned patients with atrial fibrillation on the ECG (7/47, 15%), of which three patients showed atrial fibrillation in combination with other non-acute abnormalities (non-specific ST abnormalities or left fascicular block) (see Supplementary material online, *Figure S5*) and the last case (1/47, 2%) represented a paced rhythm (see Supplementary material online, *Figure S6*).

With the Marquette 12SL algorithm, undertriage was observed in 73 patients (6.8%) and overtriage in 155 patients (15%). The four patients with an acute final diagnosis, but misclassified as not acute, presented with pan-ischaemia (2/2, 50%) and high-degree atrioventricular (AV) block (2/2, 50%). The 13 patients with a subacute final diagnosis, but misclassified as not acute or normal, presented with a prolonged QT interval (6/13, 46%), atrial fibrillation with fast ventricular response (5/13, 38%), or pericarditis (2/13, 16%). In 62 of the 66 ECG misclassified as acute (94%), but with a final not acute diagnosis, signs of ischaemia were mentioned in the Marquette 12SL diagnosis.

### Associations with clinical care and outcomes

Overall, patients with a higher predicted triage class were more often referred for cardiac follow-up, more often diagnosed with cardiac disease, and had worse clinical outcomes (i.e. longer hospital admission and higher mortality rate; *Table 3*). Moreover, patients with a higher predicted class in general also represented patients with more severe clinical diagnoses (see Supplementary material online, *Table S1*).

**Table 3:** Differences in provided clinical follow-up, diagnosis, and outcomes per predicted triage class. A *P*-value of <0.05 denotes significant difference

	Normal	Abnormal, not acute	Abnormal, subacute	Abnormal, acute	*P*-value
	*n* = 529	*n* = 373	*n* = 29	*n* = 82	
Follow-up, *n* (%)					
Cardiologist consulted?	79 (15%)	117 (31%)	19 (66%)	54 (66%)	<0.0001*
Change in management after ECG^a^	34 (6%)	72 (19%)	17 (59%)	47 (57%)	<0.0001*
Follow-up appointment cardio clinic?	36 (7%)	80 (21%)	11 (38%)	31 (38%)	<0.0001*
Final diagnosis of cardiac disease, *n* (%)	27 (5%)	194 (52%)	24 (83%)	53 (65%)	<0.0001*
Clinical outcomes					
Length of stay, mean (SD), days	3 ± 13	4 ± 10	6 ± 13	6 ± 21	<0.0001**
Hospital mortality	3 (0.6%)	8 (2%)	0 (0%)	10 (12%)	<0.0001**

^a^Change in management is defined as either a cardiac medication change, a performed cardiac procedure, when the patient was admitted to a cardiology department, or when cardiac follow-up diagnostics (e.g. ECG/lab) were proposed.

*Pairwise comparisons showed a significant difference for all pairwise comparisons (*P* < 0.001), except for the difference between the subacute and acute group (all *P* > 0.05).

**Pairwise comparisons showed a significant difference for the normal group vs. either the abnormal not acute, subacute, or acute group (*P* < 0.001) for the in-hospital length of stay. Pairwise comparisons showed a significant difference between the normal and acute group and the abnormal not acute and acute group (*P* < 0.001) for both in-hospital and 1-year mortality.

ECG, electrocariogram; SD, standard deviation.

Of the 529 (52% of the cohort) patients with an ECG classified as normal by DELTAnet, a cardiologist was consulted in 79/529 (15%) of the cases, mostly in the ER. For most patients, this did not result in a change of management (45/529, 8.5%). For the other 34/529 (6.4%) patients, follow-up was recommended (additional diagnostics or admission to a cardiology ward) in 15/34 (44%) patients, a change in medication was made in 14/34 (41%) patients, and a cardiac procedure (percutaneous coronary intervention, coronary artery bypass grafting surgery, pericardiocentesis, or pacemaker implantation) was performed in 5/34 (15%) patients. Of these 34 patients with a change in management, 1 patient represented acute pathology (acute coronary syndrome without clear ECG abnormalities); the others all represented normal or abnormal non-acute patients. In these cases, a cardiologist was consulted based on cardiac complaints or for other questions (e.g. to evaluate possible cardiac spread of infections or help in determining the appropriate treatment plan because of a history of cardiac disease: 17/34 patients were already known with cardiac disease). All 34 cases are described in detail in Supplementary material online, *Figures S7–S40*.

Of the 82 patients with an ECG predicted as acute by DELTAnet, a cardiologist was consulted in 55/82 (67%) patients. In the 27 (34%) patients where no cardiologist was consulted, most patients (15/27, 56%) had ECG abnormalities consistent with previous ECGs and therefore did not require follow-up. These ECGs were mostly acquired for other reasons than clinical complaints: only 2/27 (7%) patients had symptoms of chest pain, while 8/27 (30%) were routine control ECGs at the POS or outpatient clinic, 3/27 (11%) were acquired to evaluate whether a medication change would be allowed (risk for long-QT abnormalities), and for the other 14/27 (52%), the reason for ECG was not documented.

## Discussion

This study is the first to prospectively assess the impact of implementing an ECG-based AI algorithm for triage of 12-lead ECGs in non-cardiology departments. We demonstrated DELTAnet to be safe when implemented in clinical practice: no important ECGs were missed, and the number of ECGs predicted as acute that did not require follow-up was very limited (2.6%). Moreover, we showed excellent classification performance for both the overall population and when stratified in subgroups, similar to the original test data set, and outperforming the currently employed Marquette 12SL algorithm.^17^ Therewith, this indicates that DELTAnet can be safely used to prioritize non-cardiology ECGs by automatically assessing normal ECGs and by potentially warning the physician for acute ECGs requiring immediate follow-up by a cardiologist.

Classification performance of DELTAnet was excellent in this prospective validation data set with an overall c-statistic of 0.96 (95% CI 0.95–0.97), comparable with the performance during internal validation [c-statistic 0.93 (95% CI 0.92–0.95)].^17^ The internal validation data set was annotated by a panel of electrophysiologists that only had access to the ECG. For some ECG abnormalities, such as wide complex tachycardia or ST-segment deviations, additional information from previous ECGs, follow-up, or additional diagnostics is needed for accurate triage. In the current validation data set, we therefore took all clinical data into account to determine the final clinical diagnosis associated with this ECG. This led to many previously acute ECGs being classified as not acute. It turns out that in the previous study, the panel labelled many ECGs with ST-segment abnormalities or wide complex tachycardia as acute when having no knowledge of the other clinical information. The reclassification in the current analysis led to an increase of the sensitivity of the acute class from 79% to 96%, without reducing specificity.

One other study investigated the use of DNNs for triage of ECGs in the emergency department and showed improved performance over a conventional rule-based ECG algorithm.^23^ Although their algorithm shows similar sensitivity and specificity for differentiating normal and abnormal ECGs, DELTAnet greatly outperforms their sensitivity in detecting acute ECGs (53% vs. 96%), making it much safer for use in clinical practice. Comparison with other studies remains challenging, as wide varieties of ECG abnormalities are assessed in different studies using different metrics. One important observation from a recent meta-analysis is, however, that non-cardiologist physicians perform poorly in interpreting ECGs with a pooled accuracy of 69%.^5^ This is exactly the area where the current algorithm can be used to prevent important ECGs from being missed while saving time by prioritizing other ECGs.

### Over- and undertriage

For DELTAnet to be safe and efficacious for implementation into clinical practice, undertriage (failure to identify patients that need to be referred) and overtriage (false alarms, unnecessary consultations of the cardiologist) should be minimized. DELTAnet showed very high negative predictive values compared with clinical triage classes for the acute classes (NPV = 0.99; *Table 2*). This is among the most important findings of the current study, as it allows for safe implementation of the algorithm in clinical practice. It must be noted that there were some cases of non–ST-elevation acute coronary syndrome and unstable angina classified as normal or not acute, but these patients did not have ECG abnormalities at the time (see Supplementary material online, *Table S1*). Therefore, one should realize that the main goal of DELTAnet is to support physicians in decision-making regarding the acuteness and prioritization of new acquired ECGs; DELTAnet does not aim to (and will not be able to) substitute clinical decision-making. Only patients with ECG abnormalities at time of ECG can be detected using such an algorithm.

Most undertriage was seen for the abnormal, not acute class, where 7.7% of ECGs with that final diagnosis were classified as normal (*Figure 2*). Detailed inspection of the cases showed that this was mostly due to disagreement between the treating physician and algorithm on the meaning of non-specific ST-segment abnormalities. In practice, in 15% of the patients with an ECG classified as normal by DELTAnet, a cardiologist was consulted, which resulted in a change of management in 6% of patients. These cases concerned patients where the clinical presentation of the patient was leading in clinical decision-making and no or minimal abnormalities were seen on the ECG. None of these patients would therefore have been wrongfully overlooked by a cardiologist if DELTAnet would have been implemented.

The main challenge of the algorithm resides in the overtriage of acute disease, showing a lower positive predictive value (0.27) for this class. This lower PPV results from weighting in the training phase, where the algorithm was penalized for undertriaging acute ECGs, as this might cause undesirable false negatives in clinical practice. The PPV in the current validation set is lower than the original study. The panel that labelled the validation data set in the original study marked many ECGs as acute based on ST-segment abnormalities. These are now classified as abnormal not acute when taking previous ECGs and other tests (such as troponin testing and the outcomes of coronary angiography) into account. This distinction between non-acute and acute ST abnormalities remains a challenge, especially as DELTAnet cannot take into account symptoms or previous ECG without a current diagnosis of ACS. Overtriage is not expected to have much negative consequences: ST abnormalities can be dangerous when undetected, so consultation with an expert to justify or rule out possible ischaemia seems appropriate in these cases. The high rate of false positives for the acute class could lead to alarm fatigue, as most of the ECGs predicted as acute do not need acute follow-up.^24^ Overall, however, these false positives only account for 5.9% of the ECGs, lowering the risks of alarm fatigue (*Figure 2*).

### Limitations

There are several limitations to address. First, the number of times a cardiologist was consulted might be underestimated, as this may not always have been logged. However, important cases are always documented so it can be assumed that when not documented, no further follow-up was required. Second, our study is a background implementation study and therefore we were not able to perform extra diagnostic tests to justify the results or perform further investigation. This could have led to an underestimation of undertriage of the acute class, as patients with acute myocardial ischaemia could have been missed completely. Also, we are not able to assess the effect that implementation of DELTAnet would have on clinical decision-making, despite the prospective nature of this study. Third, electrode configuration (e.g. standard or Mason–Likar) was not available in our data set and we could therefore not evaluate the influence of different configurations on the performance of the algorithm. The Mason–Likar configuration is mostly used in the emergency department, and our subgroup analyses do not show a difference in performance in that subgroup, suggesting that the electrode configuration has limited influence on algorithm performance.

### Implications for future work

An important next step will be to perform a randomized controlled trial to evaluate implementation in real-world clinical practice with its true impact on clinical care and patient outcomes. Other future perspectives to improve its clinical applicability include adding visualization methods and uncertainty models that can identify the cases the algorithm is prone to misdiagnose.^25,26^ In addition, another future goal is to investigate whether automated comparison of a new acquired ECG with previous ECGs would be possible. Eventually, an ‘AI-ECG dashboard’ needs to be developed that is able to clearly present the ECG with predicted triage categories along with ECG features important for prediction. At last, a goal is to implement DELTAnet in mobile ECG devices, making it applicable for use in pre-hospital settings or places where standard 12-lead ECG is not readily available.

## Conclusions

This study is the first to prospectively validate an ECG-based AI triage algorithm and provide insight into its clinical implementation. We demonstrated that DELTAnet is safe to be used in clinical practice for triage of 12-lead ECGs, acquired at non-cardiology departments, and outperformed the currently employed algorithm for computerized interpretation of the ECG (Marquette 12SL). Implementation of DELTAnet could possibly lead to decreased workload for physicians and quicker recognition of acute life-threatening cardiac disorders. As a next step, a randomized study will be performed to evaluate its added value on clinical care and patient outcomes compared with current care.

## Supplementary material

Supplementary material is available at *European Heart Journal – Digital Health*.

## Supplementary Material

ztad070_Supplementary_Data

## Data Availability

The data sets used in this study are not openly available due to privacy concerns.
