# Supplementary material for: Automatic triage of twelve-lead electrocardiograms using deep convolutional neural networks: a first implementation study
Source: Eur Heart J Digit Health. 2023 Nov 8;5(1):89–96. doi: 10.1093/ehjdh/ztad070 (PMC10802816; doi:10.1093/ehjdh/ztad070)
Supplement: ztad070_Supplementary_Data [file ztad070_supplementary_data.docx]

**Supplemental Material**

**Supplemental Table 1**

|  | Normal  (n = 529) | Abnormal  not acute  (n = 373) | Abnormal sub-acute  (n = 29) | Acute  (n = 82) |
| --- | --- | --- | --- | --- |
| **Ischemia, n (%)** |  | | | |
| ACS NSTEMI |  | 7 |  | 7 |
| Unstable AP | 1 | 2 |  |  |
| Post cardiac arrest |  | 1 |  | 4 |
| Old MI (e.g. pathological Q's) | 7 | 36 |  | 1 |
| **Rhythm disorders, n (%)** |  | | | |
| Ventricular tachycardia |  |  |  | 2 |
| AV(N)RT |  |  |  | 5 |
| Junctional escape rhythm |  |  |  | 1 |
| Bradycardia < 50/min | 6 | 6 |  |  |
| Atrial flutter |  | 1 | 8 | 4 |
| Atrial fibrillation > 100/min |  |  | 14 | 3 |
| Atrial fibrillation < 100/min |  | 12 |  | 5 |
| Paced rhythm |  | 21 |  | 2 |
| Undefined rhythm |  | 1 |  |  |
| **Conduction disorders, n (%)** |  | | | |
| 3^rd^ degree AV block |  |  |  | 1 |
| Long QTc >500ms |  | 5 | 4 | 2 |
| Borderline long QTc >470ms |  | 5 | 1 |  |
| LBBB/RBBB | 1 | 46 |  | 1 |
| Other blocks |  | 18 |  |  |
| **Infections, n (%)** |  | | | |
| Pericarditis |  |  |  | 2 |
| Endocarditis | 1 |  |  |  |
| Myocarditis |  | 1 |  | 1 |
| **Cardiomyopathy, n (%)** |  | | | |
| Ventricular hypertrophy | 4 | 21 |  | 4 |
| Atrial enlargement |  | 8 |  |  |
| Arrhythmogenic cardiomyopathy |  |  |  | 1 |
| **Other diagnoses, n (%)** |  | | | |
| Pericardial injury | 2 |  |  |  |
| Microvoltages | 3 | 12 |  |  |
| ST-abnormalities without final cardiac diagnosis | 19 | 67 | 1 | 18 |
| Left heart axis |  | 17 |  |  |
| **Multiple diagnoses, n (%)** |  | | | |
| NSTEMI + 3^rd^ AV block |  |  |  | 1 |
| Old MI + BBB |  | 3 |  |  |
| Old MI + LVH |  | 4 |  |  |
| Old MI + ST-abn. |  | 6 |  |  |
| Old MI + microvoltages |  | 4 |  |  |
| BBB + LVH |  | 4 |  |  |
| BBB + ST-abnormalities |  | 2 |  | 2 |
| AF < 100/min + left anterior fascicle block + ST-abn. |  |  |  | 1 |
| AF + bradycardia < 50/min + ST-abn. |  |  |  | 1 |
| AF > 100/min + ST-abn. |  |  |  | 2 |
| AF < 100/min + ST-abn. |  | 4 |  | 4 |
| AF < 100/min + BBB |  | 2 |  |  |
| AF < 100/min + BBB + ST-abn. |  |  |  | 2 |
| AF < 100/min + old MI |  | 3 |  |  |
| AF < 100/min + old MI + ST-abn. |  | 1 |  |  |
| Atrial tachycardia + pacing + BBB |  | 1 |  |  |
| Biatrial dilatation + ST-abn. |  |  |  | 1 |
| Microvoltages + ST-abn. | 1 | 5 |  |  |
| Microvoltages + ST-abn. + old MI |  | 1 |  |  |
| Borderline QTc + ST-abn. |  | 2 |  | 2 |
| **Non-cardiac diagnoses, n (%)** | 483 | 45 | 1 | 2 |

Abbreviations: ACS = acute coronary syndrome, AF = atrial fibrillation, AV(N)RT = atrioventricular (nodal) re-entry tachycardia, LBBB/RBBB = left/right bundle branch block, LVH = left ventricular hypertrophy MI = myocardial infarction, NSTEMI = non-ST-segment elevation myocardial infarction, ST-abn. = (non-specific) ST-abnormalities. The table only represents patients with one final diagnosis or one diagnosis in the highest category present. Patients with multiple diagnoses in one category (e.g. ECGs with both atrial fibrillation and LBBB) were not included in this sub-analysis, because it would be unclear where the algorithm bases its prediction on.

**
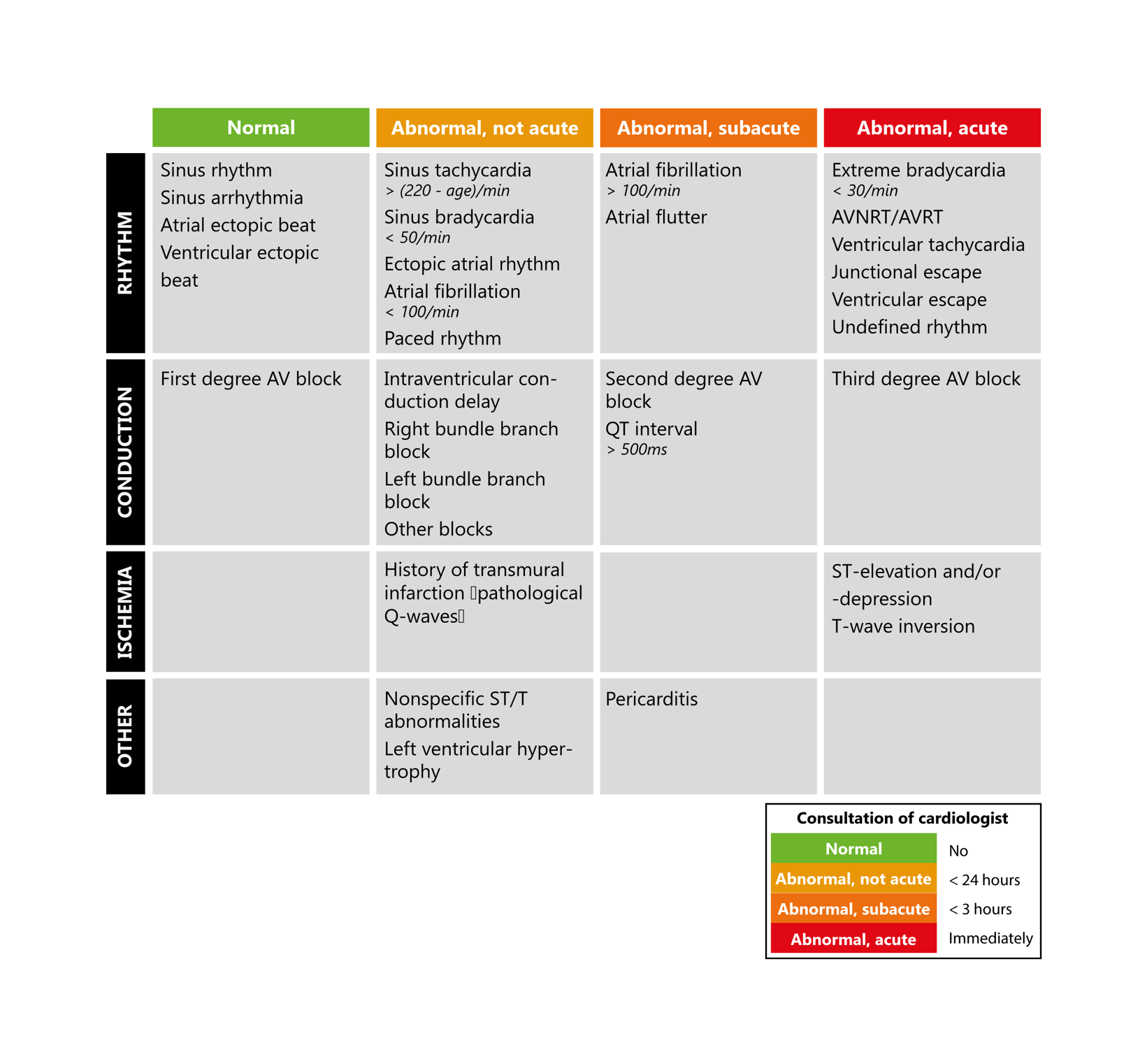
**

**Supplemental Figure 1.** ECG diagnoses with their classification in corresponding triage classes. Abbreviations: AV(N)RT = Atrioventricular (nodal) reentry tachycardia, AV block = atrioventricular block, LBBB/RBBB = left/right bundle branch block.


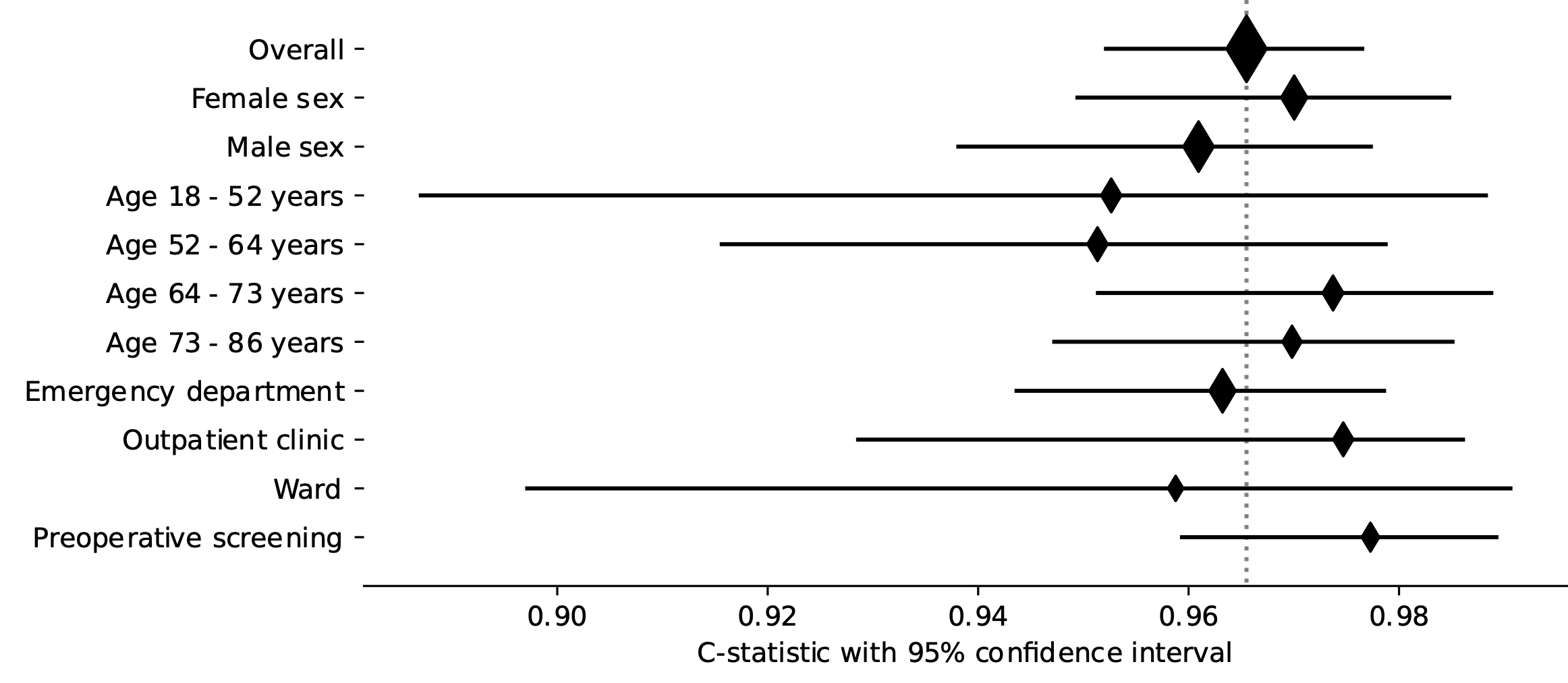


**Supplemental sFigure 2.** C-statistics (concordance statistics) comparing predicted classes clinical triage classes, stratified by age, sex and hospital location. Performance metrics are shown with corresponding 95% confidence interval.


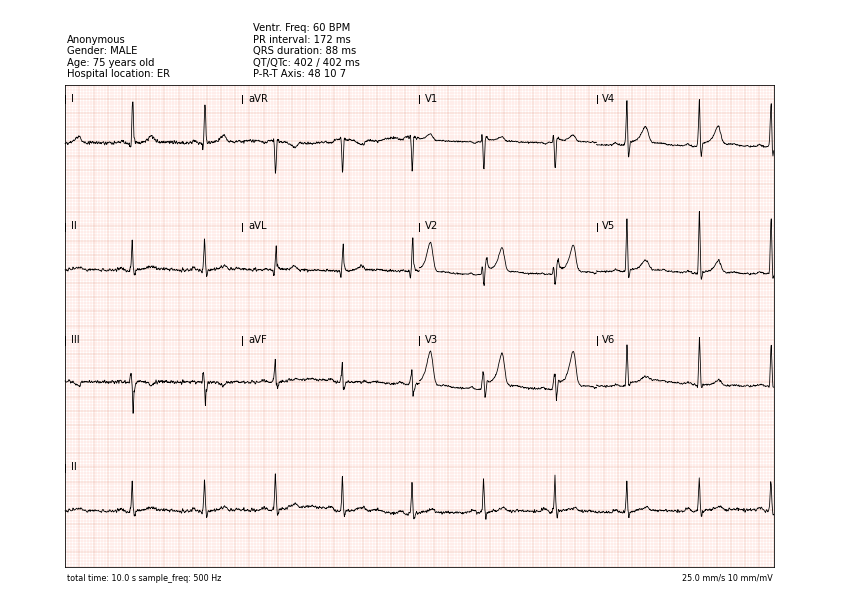
**Supplemental Figure 3.** Example case of a clinical acute patient predicted to be non-acute by DELTAnet. The patient was clinically triaged as acute because of a final diagnosis of unstable angina pectoris and visible ST-wave abnormalities at time of ECG. The ECG shows isolated concave ST-elevation in lead V2, V3 without reciprocal ST-depression in other leads. The consulted cardiologist documented that at time of ECG the patient was not primary suspected for ischemia (normal lab/echo, not a typical ischemia ECG). When other diagnoses were ruled out, a coronary angiogram was performed, which confirmed the presence of significant coronary artery disease.


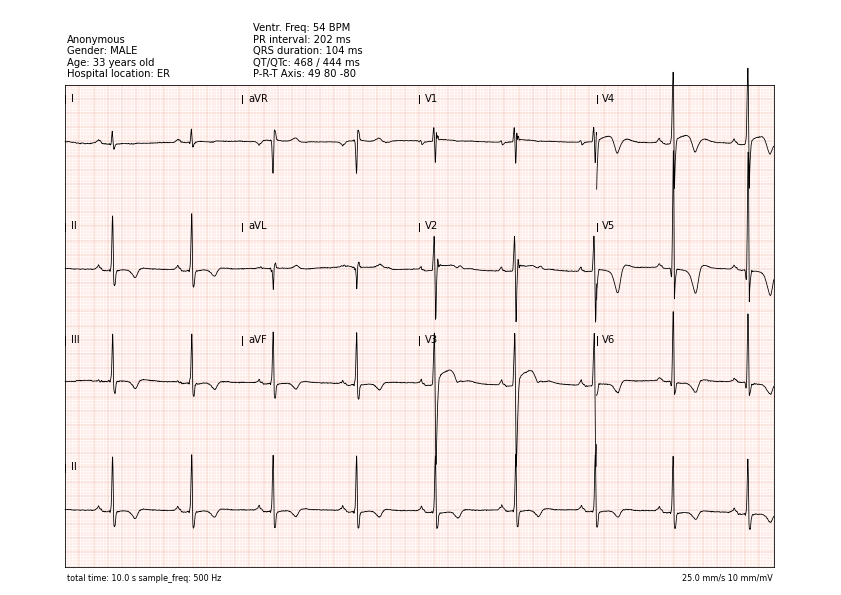

**Supplemental Figure 4:** Example case of a patient predicted to be acute by DELTAnet. The ECG shows ST-abnormalities in V2-V5 and deep negative T-waves inferior (comparable to previous ECG except for deeper T-waves). An acute cardiac syndrome was ruled out and the patient was referred to a cardiology clinic for follow-up appointment. The patient was clinically classified as non-acute, because of the presence of non-acute cardiac disease (cardiomyopathy).


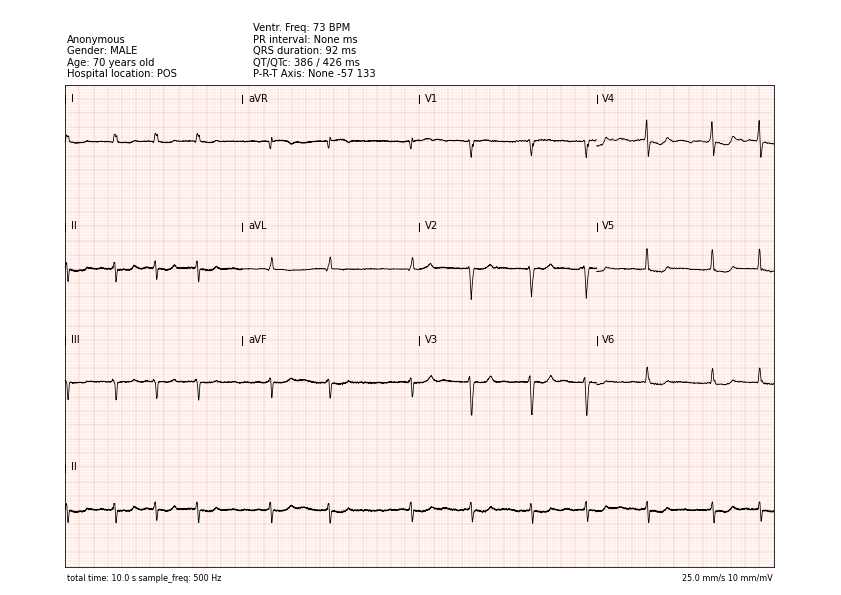

**Supplemental Figure 5:** Example case of a patient predicted to be acute by DELTAnet. The ECG shows atrial fibrillation, lateral repolarization abnormalities, and left anterior fascicle block (ECG was non-dynamic compared to previous ECGs). Patient was clinically classified as non-acute, because of the presence of non-acute cardiac disease (atrial fibrillation).


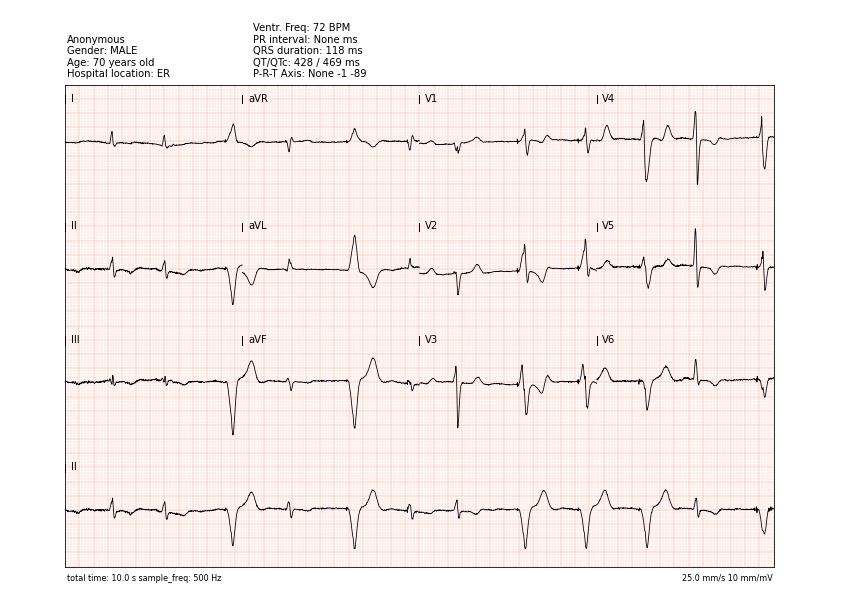
 **Supplemental Figure 6:** Example case of a patient predicted to be acute by DELTAnet. The ECG shows ventricular pacing with frequent ventricular ectopic beats, but also own cardiac activity with a very long PQ-time. Patient was clinically classified as non-acute, because of the presence of non-acute cardiac disease (paced rhythm).


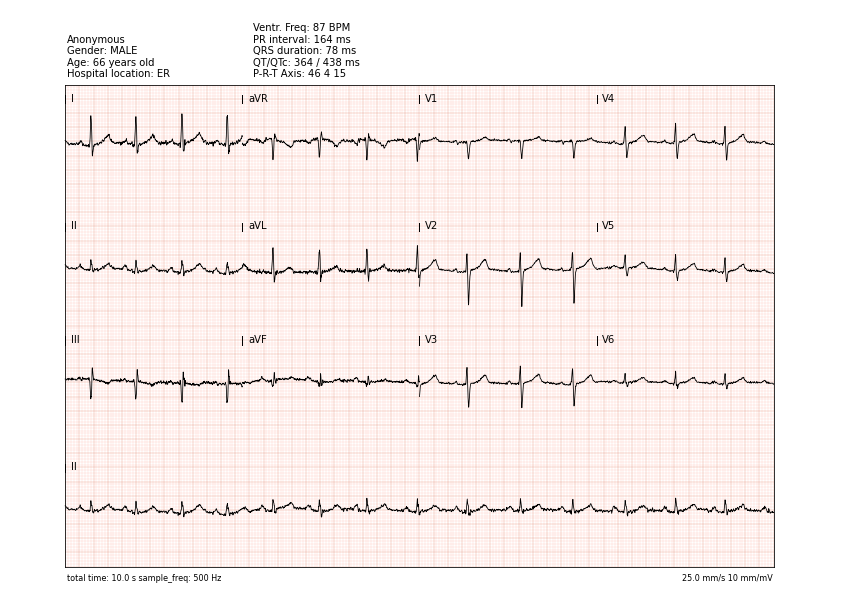
 **Supplemental Figure 7:** Example case of a normal predicted ECG by DELTAnet. The ECG shows sinus rhythm and no other abnormalities. A cardiologist was consulted because of chest pain complaints and dyspnea on exertion. The patient was admitted to the CCU, where rhythm observation was performed. The patient had no final cardiac diagnosis and no further follow-up was required.


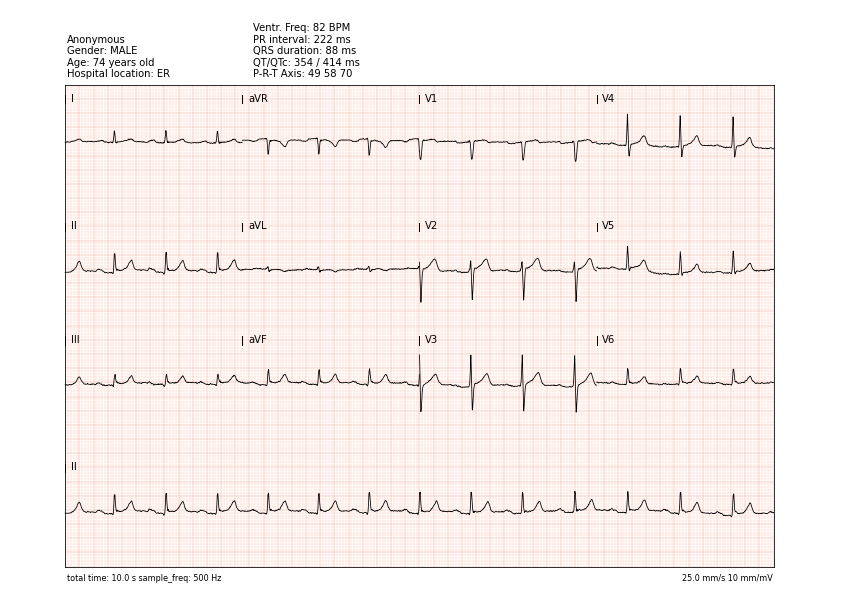


**Supplemental Figure 8:** Example case of a normal predicted ECG by DELTAnet. The ECG shows sinus rhythm with a flat T in aVL and subtle ST-elevation in II, III, aVF, V5-V6, most suspected for early repolarization. The cardiologist was consulted for help in ECG interpretation, because of a suspicion for ischemia as based on a passage of palpitations complaints during exercise. The cardiologist mentioned the ECG to be most suggestive for early repolarization (but because of the flat T in aVL to be sure requested troponin lab as follow-up). Because serial troponin measuements were normal, no further follow-up was required and there was no change in management.


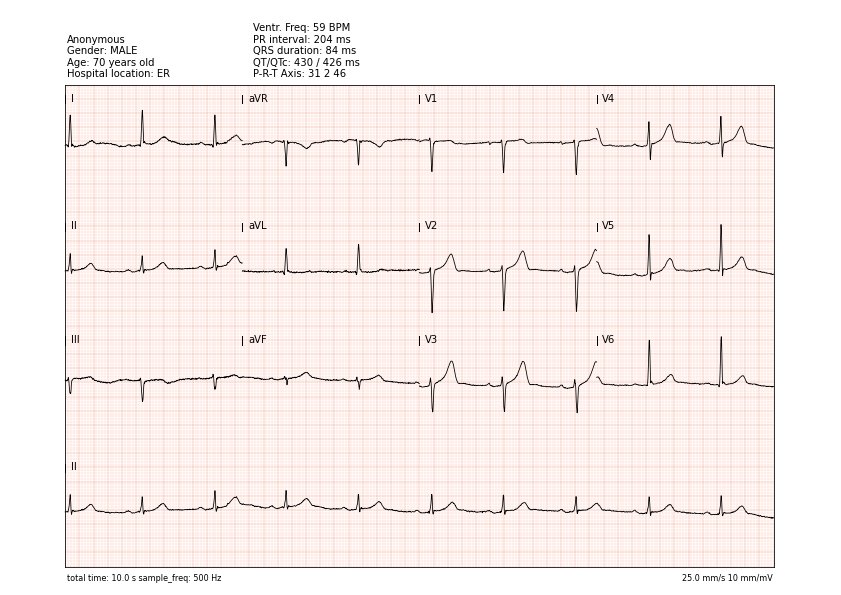
 **Supplemental Figure 9:** Example case of a normal predicted ECG by DELTAnet. The ECG shows sinus rhythm with flat repolarization in aVL and minimal diffuse ST-elevation. The cardiologist was consulted with the question to assess the ECG for right heart failure/stress because the patient was diagnosed with pulmonary embolism. The cardiologist concluded that there were no signs of right heart failure and that the subtle ST-elevation could be caused by pericardial irritation. As the patient was suspected for pericardial irritation (symptoms in combination with the subtle ST-abnormalities), a new ECG for the next morning was advised. No other change in medical policy occurred and the patient had no final diagnosis of cardiac disease.


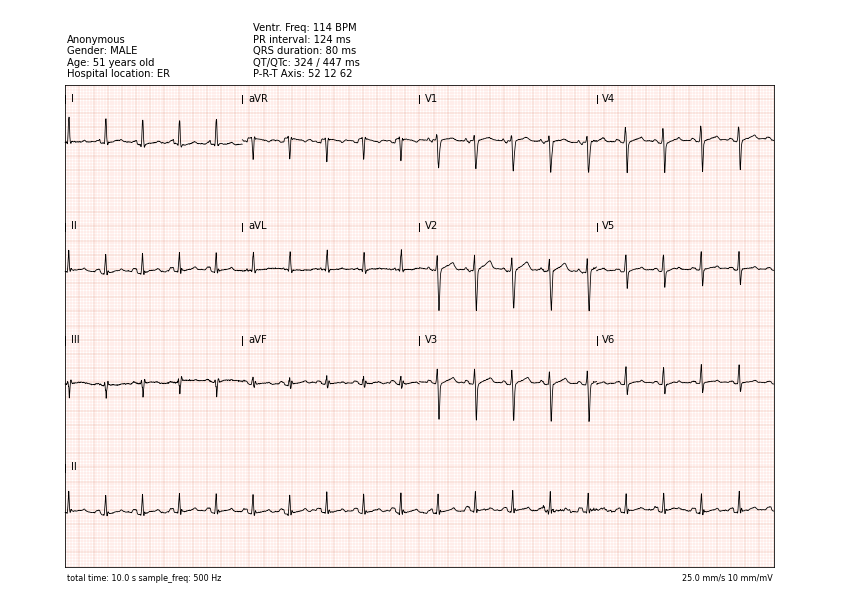
 **Supplemental Figure 10:** Example case of a normal predicted ECG by DELTAnet. The ECG shows sinus tachycardia without ECG abnormalities. The cardiologist was consulted because of pericardial effusion that was visible on CT. The cardiologist concluded that there were both no visible ECG abnormalities and symptoms that would be indicative for hemodynamically important pericardial effusion or pericarditis. Therefore, an conservative treatment plan was advised. After a few days, complete recovery from the pericardial effusion was seen. No other change in medical policy occurred, no final diagnosis of cardiac disease.


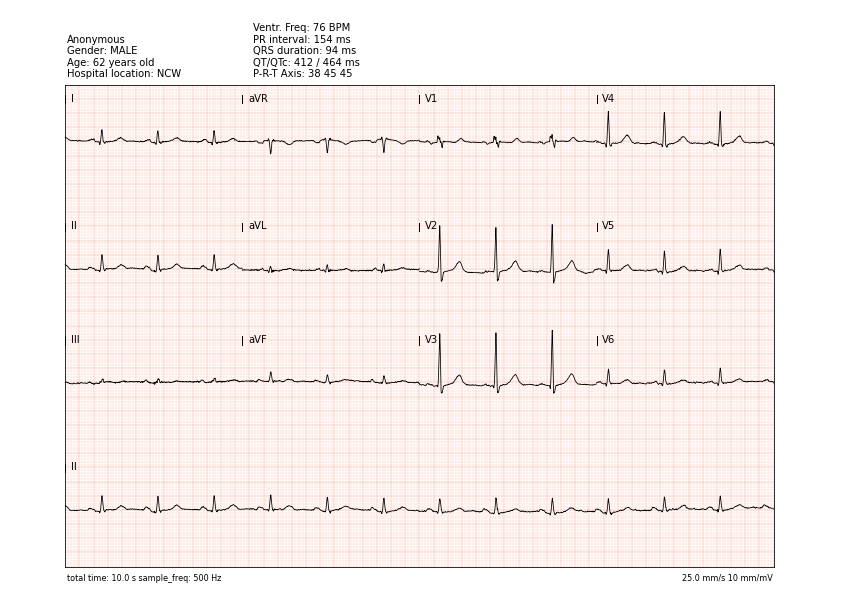
 **Supplemental Figure 11:** Example case of a normal predicted ECG by DELTAnet. The ECG shows sinus rhythm with early R-wave progression. The cardiologist was consulted because of S. Aureus bacteremia with unclear focus and possible diagnosis of endocarditis. The cardiologist concluded that there were no visible ECG abnormalities indicative for endocarditis, but follow-up (ECG, transthoracic echocardiogram) was recommended to keep track on possible later cardiac involvement. The patient was diagnosed with an endocarditis 14 days after above ECG was acquired.


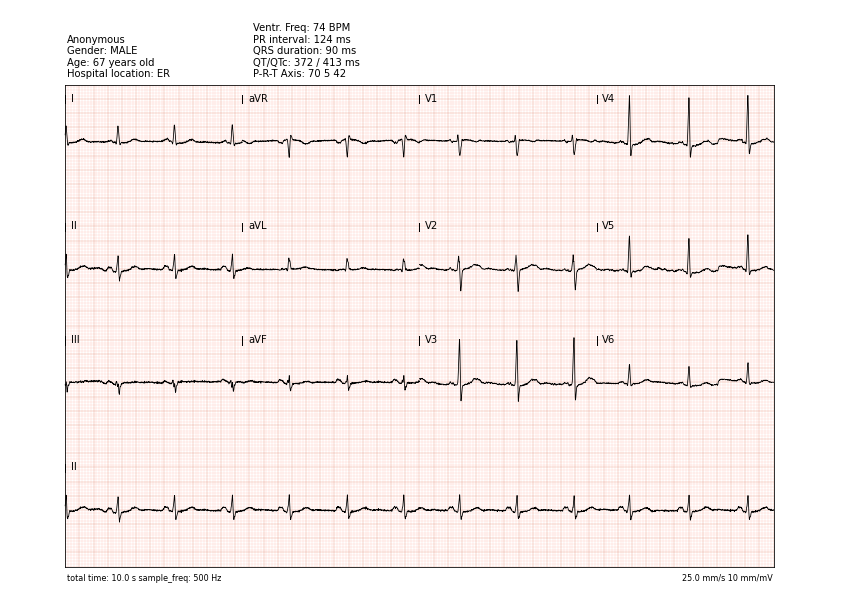
 **Supplemental Figure 12:** Example case of a normal predicted ECG by DELTAnet. The ECG shows normal sinus rhythm. Patient was admitted because of a sudden syncope. A cardiologist was consulted to evaluate whether this could be caused by a cardiac disorder. There were yet no signs for a cardiac cause; however cardiac follow-up (lab) was recommended to be sure. Patient had no final diagnosis of cardiac disease.


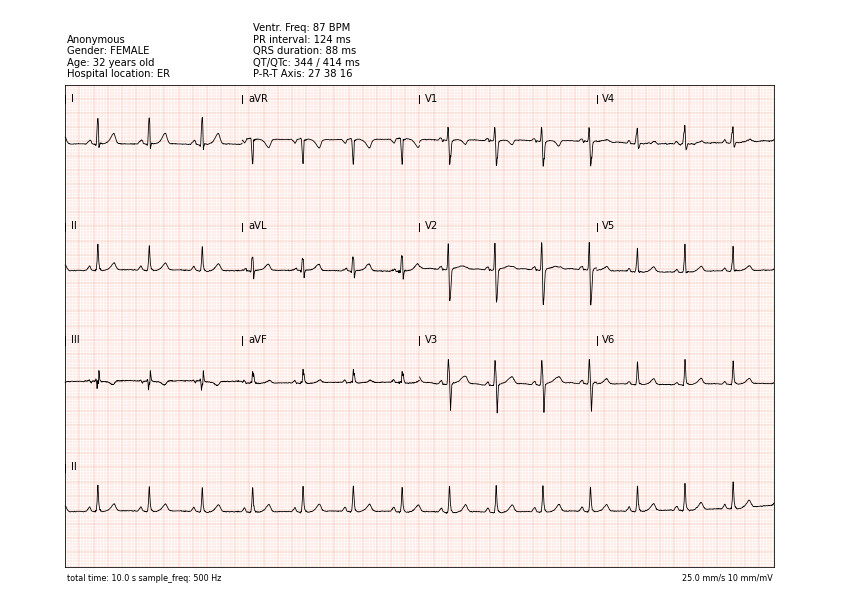
 **Supplemental Figure 13:** Example case of a normal predicted ECG by DELTAnet. The ECG shows normal sinus rhythm. The cardiologist was consulted because of chest pain complaints after trauma and concluded that there were no signs for heart contusion. Cardiac follow-up was recommended (lab), with the request to only consult a cardiologist again when elevated. The patient had no final cardiac diagnosis and was discharged home the same day.


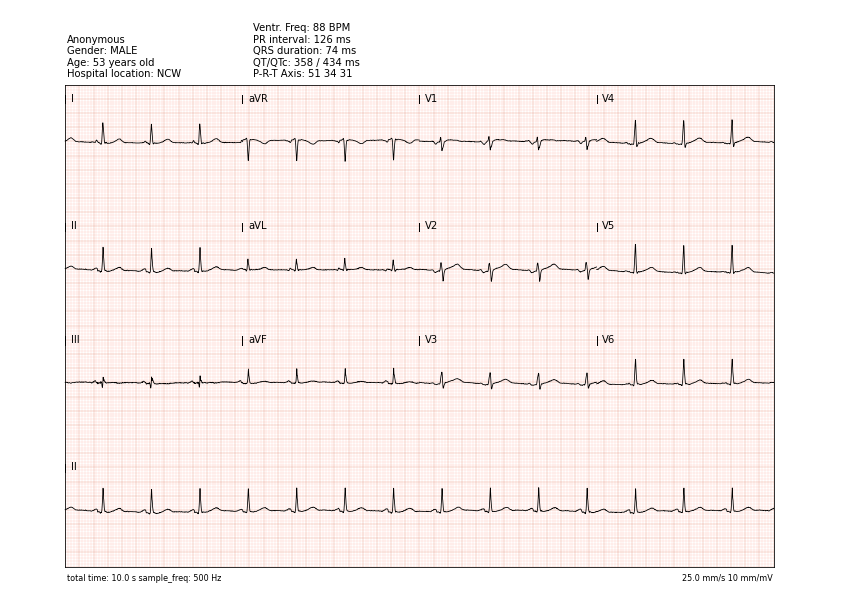
 **Supplemental Figure 14:** Example case of a normal predicted ECG by DELTAnet. The ECG shows normal sinus rhythm. Patient was admitted because of a cerebrovascular accident; the cardiologist was consulted for help in determining the appropriate treatment plan, because the patient recently underwent heart transplantation. Follow-up ECG was recommended. Patient received no new cardiac diagnosis.


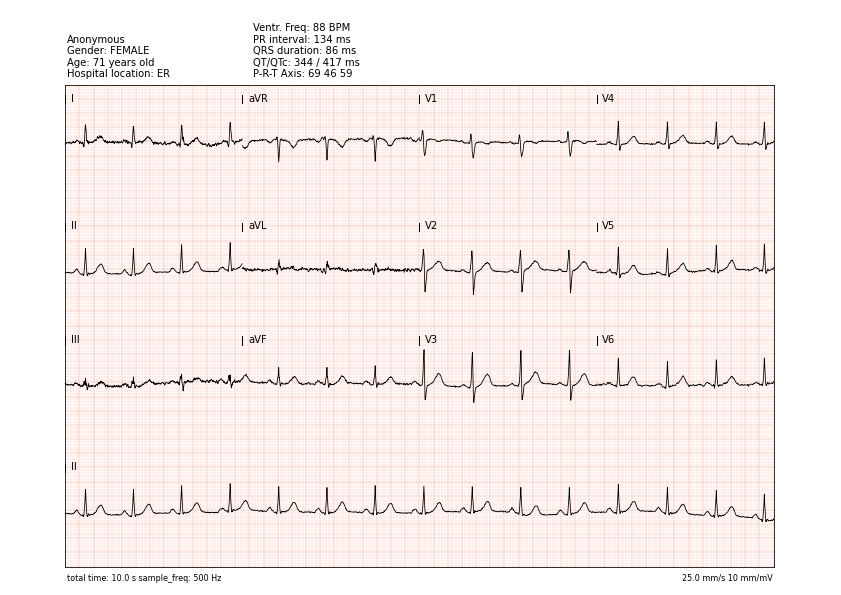
 **Supplemental Figure 15:** Example case of a normal predicted ECG by DELTAnet. The ECG shows normal sinus rhythm. The ECG was acquired because of S. Aureus infection and rule out conduction abnormalities associated with endocarditis. The consulted cardiologist concluded there were no signs for endocarditis at this point in time, but recommended follow-up with echocardiogram? And ECG for follow-up. There were no signs of endocarditis during follow-up , and patient was discharged the next day.


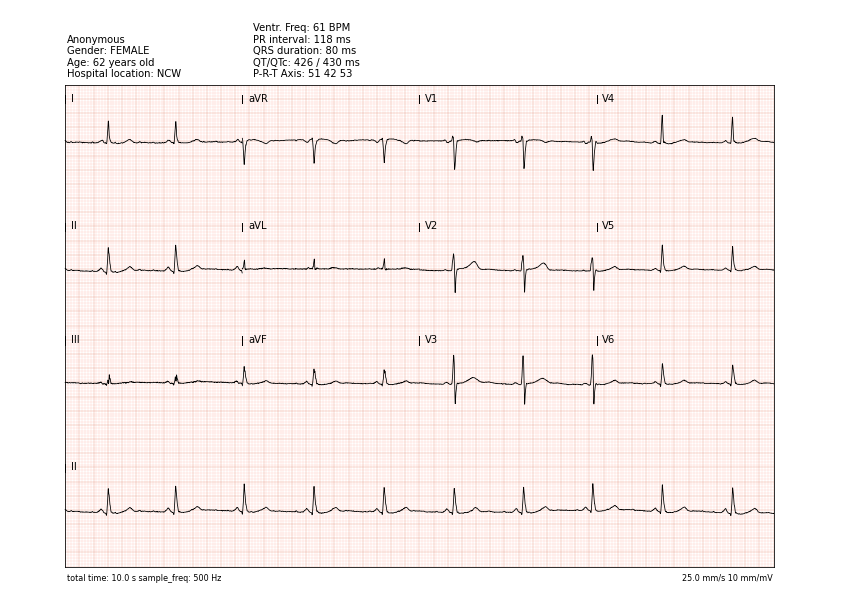
 **Supplemental Figure 16:** Example case of a normal predicted ECG by DELTAnet. The ECG shows normal sinus rhythm. The patient was admitted because of a trauma accident and showed high levels of troponin, for which a cardiologist was consulted. The cardiologist concluded there were no signs of cardiac pathology but recommended 24 hours rhythm observation, on which no abnormalities were seen. No final cardiac diagnosis was made, and the patient was discharged home the next day.


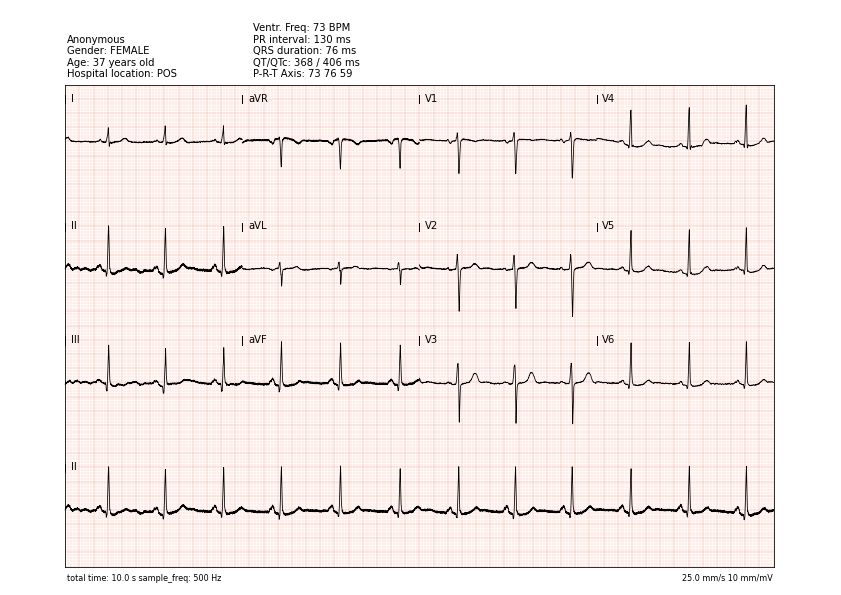


**Supplemental Figure 17:** Example case of a normal predicted ECG by DELTAnet. The ECG shows normal sinus rhythm. The cardiologist was consulted, because of a murmur on auscultation at the pre-operative screening department. Cardiac follow-up (echocardiogram) was scheduled for the next day.


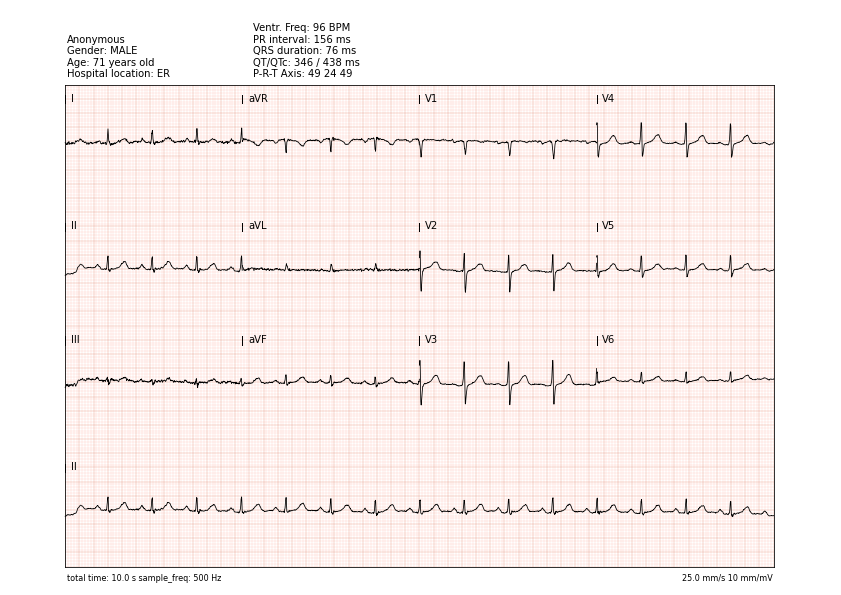
 **Supplemental Figure 18:** Example case of a normal predicted ECG by DELTAnet. The ECG shows normal sinus rhythm. The cardiologist was consulted, because of transient loss of consciousness with unknown reason. At moment of consultation, there were no signs for a cardiac pathology. However, rhythm observation until the next morning was recommended because of the patient’s history of myocardial infarction. No abnormalities were observed and the patient was discharged the next morning.


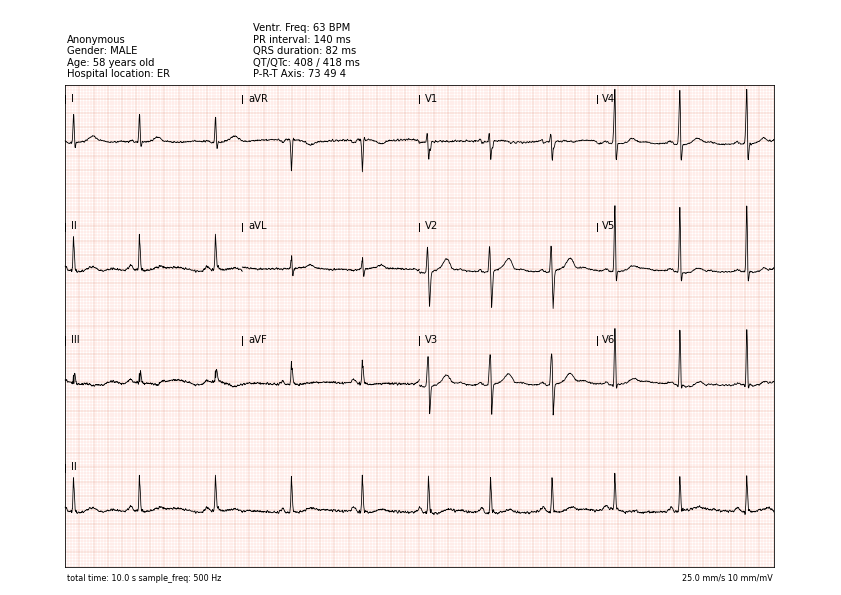
 **Supplemental Figure 19:** Example case of a normal predicted ECG by DELTAnet. The ECG shows normal sinus rhythm with minimal upsloping ST-depression in V4-V6, flat T in aVF, and borderline criteria for left ventricular hypertrophy. The cardiologist was consulted because of trauma with possible cardiac contusion and recommended cardiac follow-up (rhythm observation, lab, ECG). The patient was discharged the next day.


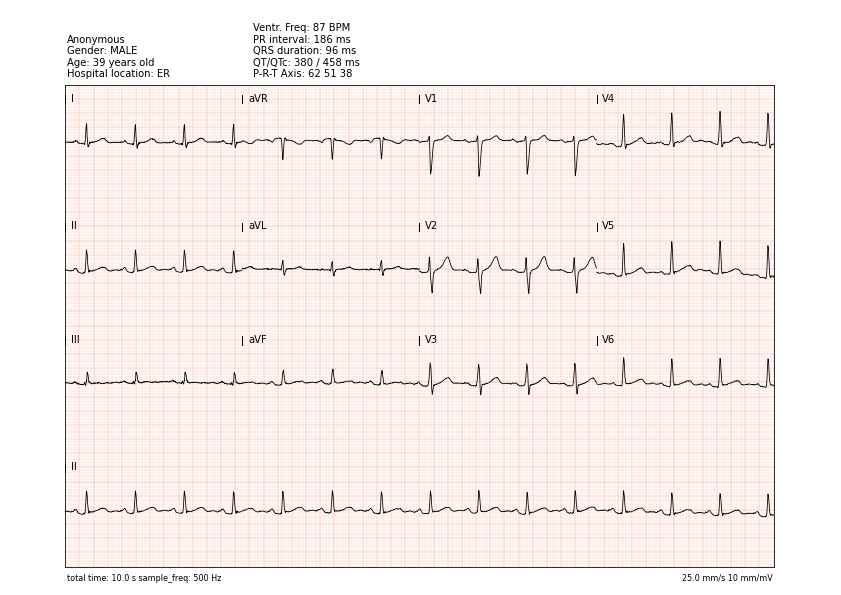
 **Supplemental Figure 20:** Example case of a normal predicted ECG by DELTAnet. The ECG shows normal sinus rhythm with (minimal) diffuse pTA depression, ST-elevation and pTA elevation in aVR. The cardiologist was already consulted because of trauma with thoracic injury. The ECG suggests pericardial injury. However, on CT there was no effusion visible and first troponin lab was negative. Cardiac follow-up (lab, ECG and when needed echocardiogram) was recommended, but did not show signs of heart contusion. No final diagnosis of cardiac disease was made.


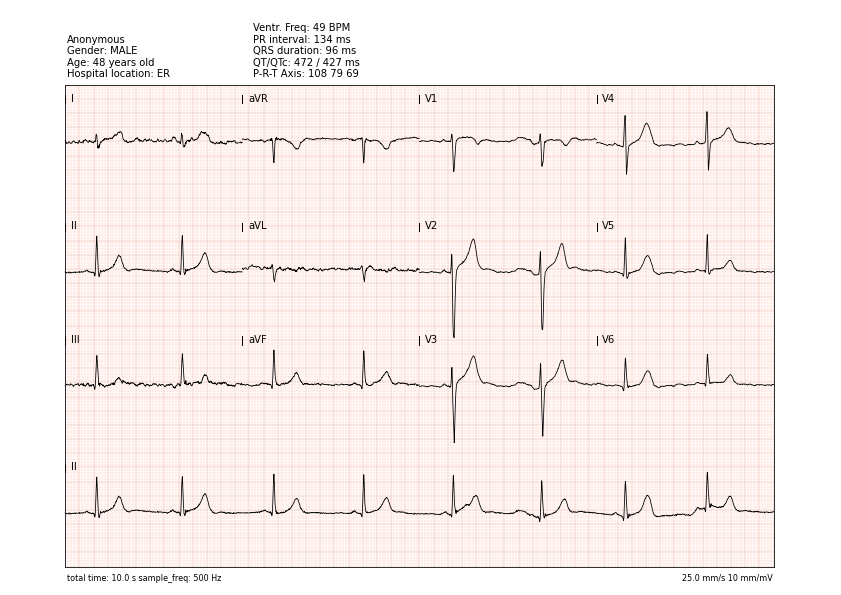
 **Supplemental Figure 21:** Example case of a normal predicted ECG by DELTAnet. The ECG shows sinus bradycardia with minimal inferior ST-elevation; not primary suspected for ischemia. Cardiologist was already consulted before ECG, because of chest pain complaints. At that moment, there were no signs for acute cardiac disease, but cardiac follow-up was recommended (ECG/lab/ergometry), to rule-out unstable AP. No cardiac cause was found and the patient was discharged the next day.


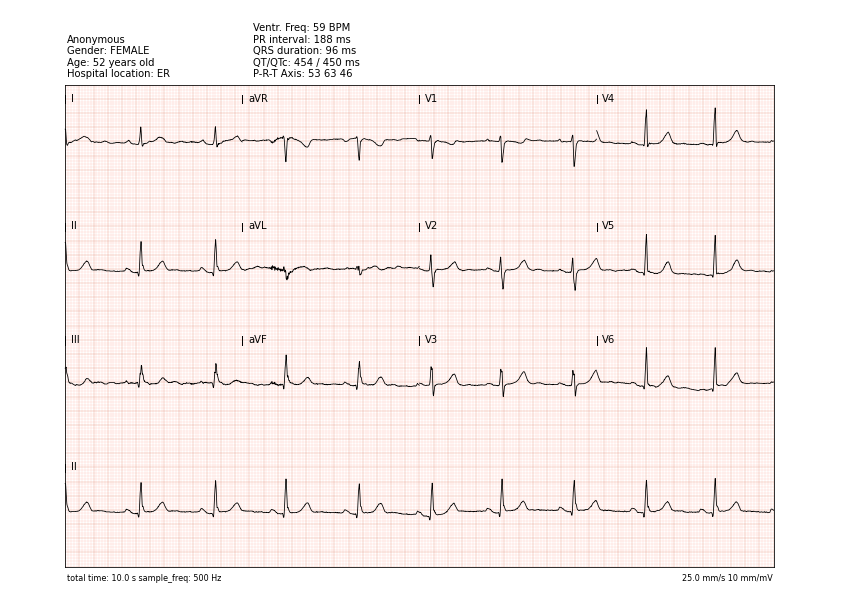
 **Supplemental Figure 22:** Example case of a normal predicted ECG by DELTAnet. The ECG shows normal sinus rhythm without abnormalities. Cardiologist was consulted because of chest pain complaints (however normal ECG and lab, therefore no ACS). Patient also had complaints of drowsiness, which was referred to be caused by the nitroglycerin patches the patient was using. Therefore, the dose of this medication was lowered to reduce side effects. Patient was discharged and no further follow-up was necessary.


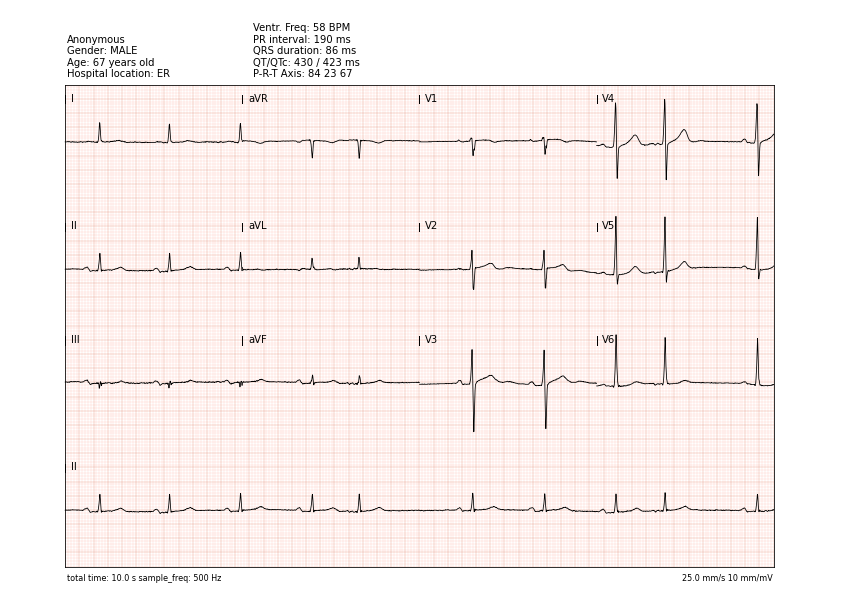
 **Supplemental Figure 23:** Example case of a normal predicted ECG by DELTAnet. The ECG shows normal sinus rhythm with few atrial ectopic beats and flat repolarization. Cardiologist was consulted because of chest pain complaints (however normal ECG and lab, therefore no ACS). The patient collapsed at the ER with bradycardia and a hypotension. Therefore, metoprolol (beta blocker) medication was reduced in dose. Patient was discharged home. Follow-up appointment at a cardiology clinic was already scheduled, because of the patient's history (myocardial infarction, atrial fibrillation).


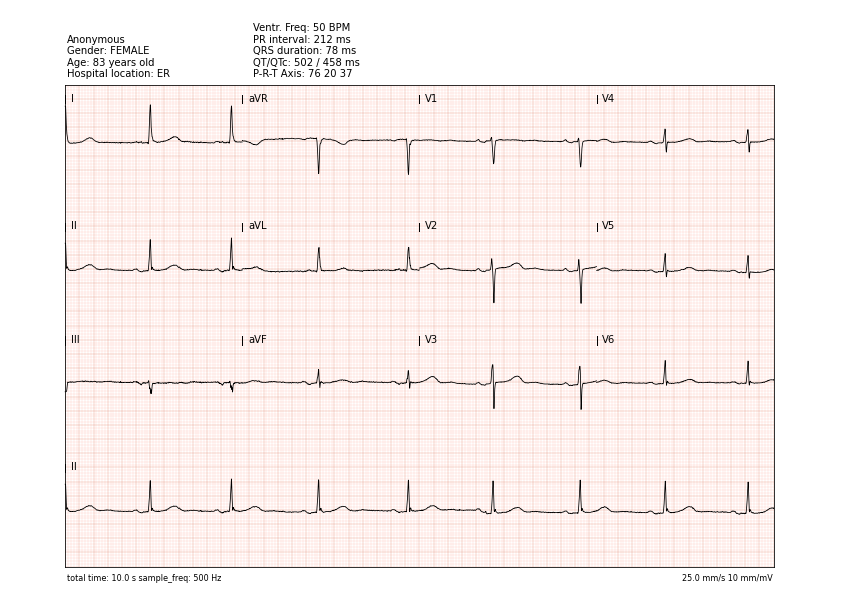
 **Supplemental Figure 24:** Example case of a normal predicted ECG by DELTAnet. The ECG shows normal sinus rhythm with 1^st^ degree AV block, no other abnormalities. The cardiologist was consulted because the patient collapsed and had symptoms of general malaise. Because of the syncope which was deemed to be caused by bradycardia and hypotension, medication dose of Bisoprolol (beta blocker) was reduced. Patient was discharged home, no further cardiac follow-up was required.


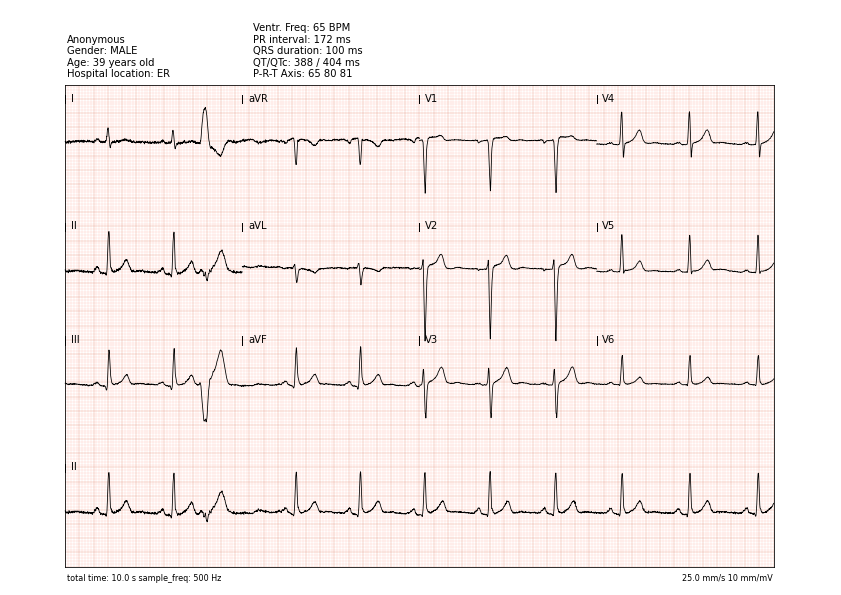
 **Supplemental Figure 25:** Example case of a normal predicted ECG by DELTAnet. The ECG shows sinus rhythm with a ventricular ectopic beat and diffuse ST-elevations strongly suggestive for early repolarization, however the negative T in aVL was more pronounced than on earlier ECGs. The cardiologist was already consulted before ECG, because of chest pain complaints. Conclusion cardiologist: aspecific chest pain complaints, possibly stress-related. Extra antilipaemics were started and the patient was discharged home the same day.


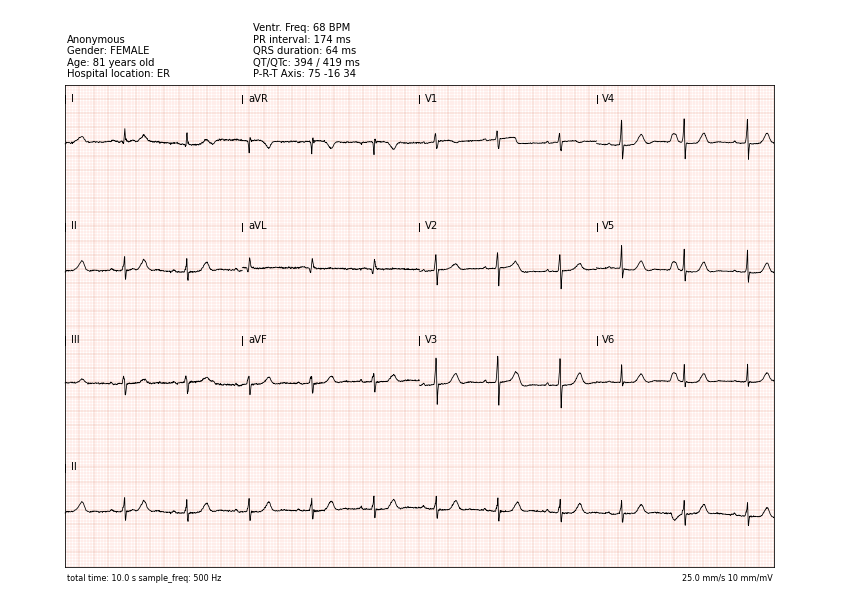
 **Supplemental Figure 26:** Example case of a normal predicted ECG by DELTAnet. The ECG shows normal sinus rhythm without abnormalities. The cardiologist was consulted because the patient came in with chest pain complaints and ischemic changes on the ambulance ECG. Complaints were attributed to an episode of transient severe hypertension because of medication use (perindopril). The patient's medication was changed, and the patient was discharged home, no further cardiac follow-up was required.


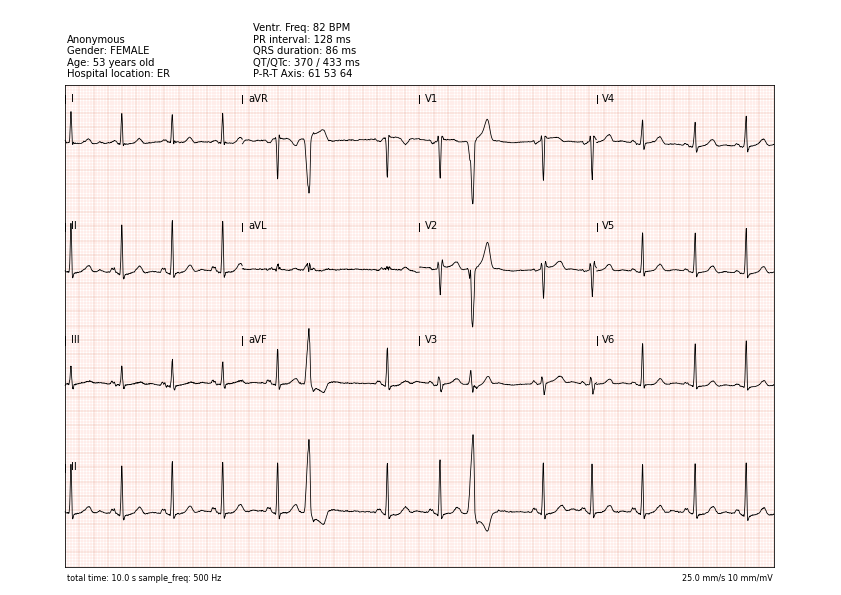
 **Supplemental Figure 27:** Example case of a normal predicted ECG by DELTAnet. The ECG shows normal sinus rhythm with two ventricular ectopic beats. The cardiologist was consulted because of palpitations complaints, which concluded the complaints to be caused by the ventricular ectopic beats. New medication was started and the patient was discharged home, no further cardiac follow-up was required.


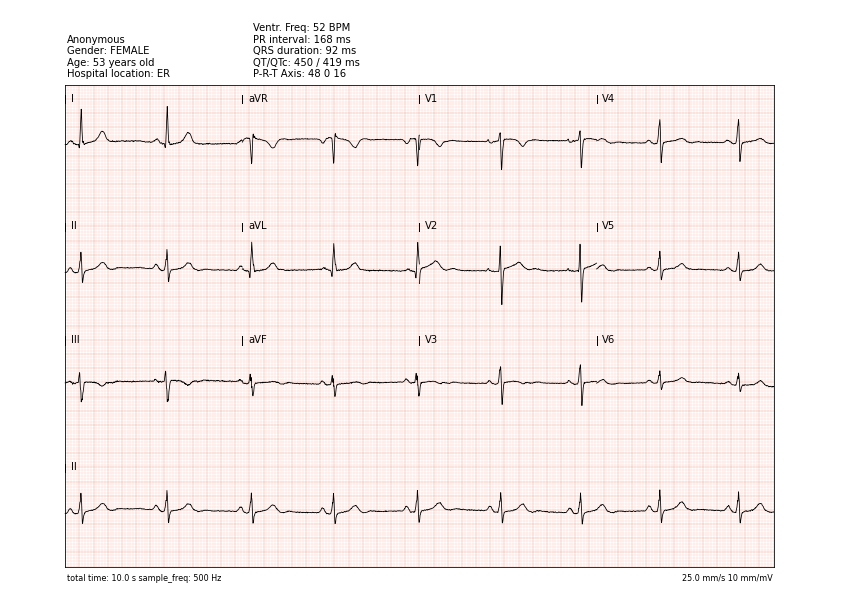
 **Supplemental Figure 28:** Example case of a normal predicted ECG by DELTAnet. The ECG shows bradycardia (52/min) with no other abnormalities. The cardiologist was consulted because of (atypical) chest pain complaints, but no cardiac explanation was found. New medication was started, and the patient was discharged home, no further cardiac follow-up was required.


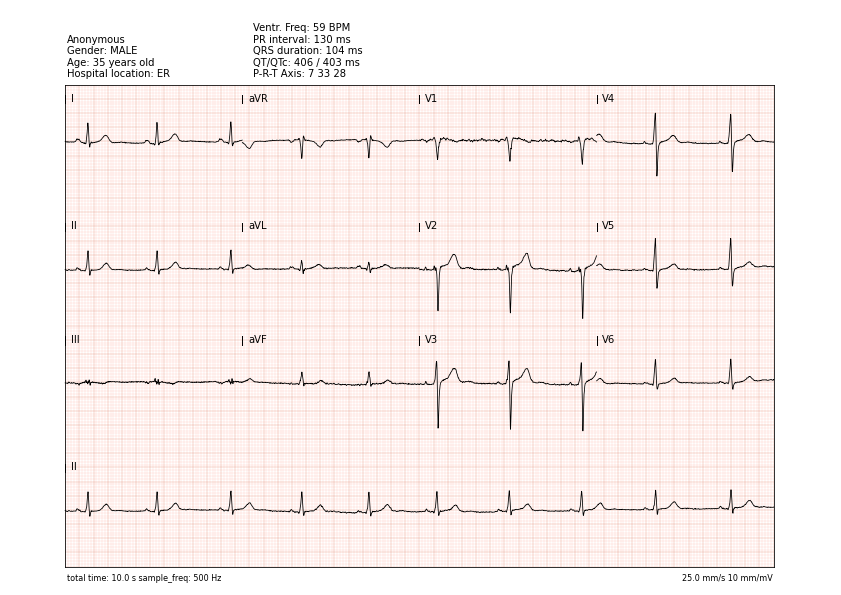
 **Supplemental Figure 29:** Example case of a normal predicted ECG by DELTAnet. The ECG shows normal sinus rhythm, no abnormalities. Cardiologist was consulted because of chest pain complaints. Complaints were determined to be atypical and attributed to hypertension. Medication was changed and a follow-up appointment at a cardiology clinical was already scheduled (because of patient's cardiac risk factors of high cholesterol and hypertension).


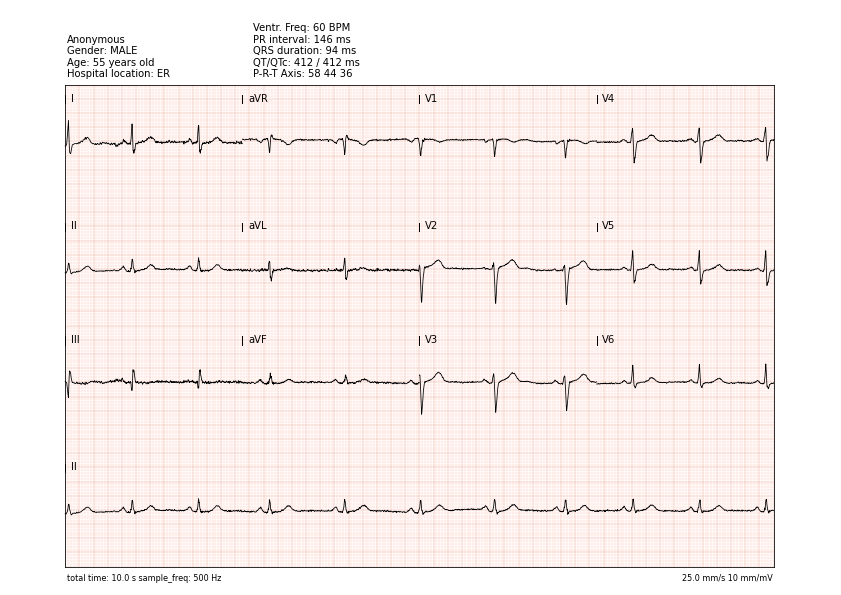
 **Supplemental Figure 30:** Example case of a normal predicted ECG by DELTAnet. The ECG shows normal sinus rhythm. The cardiologist was consulted because the patient presented with acute pain in between the scapula. Complaints were attributed to be of musculoskeletal origin, because of absence of chest pain complaints and no response to NTG. Patient was discharged home and no cardiac follow-up was required.


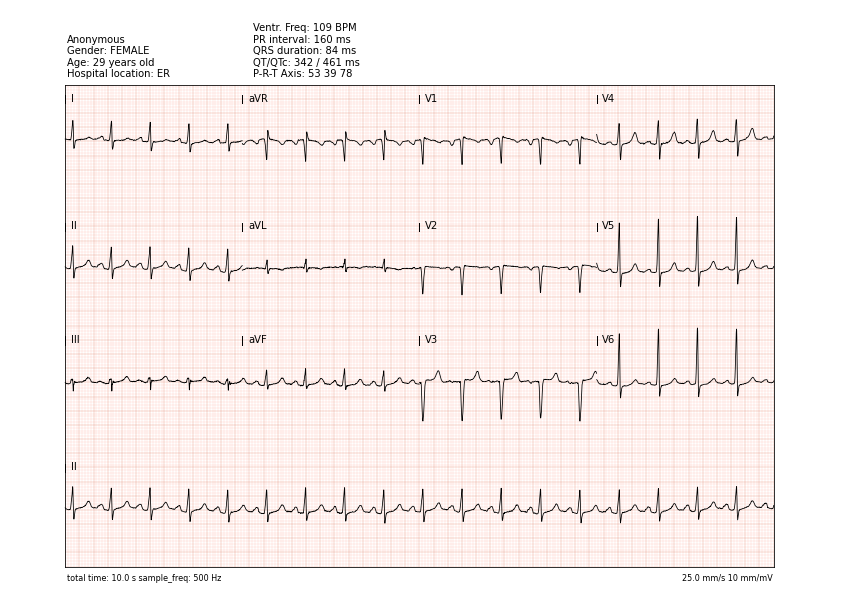
 **Supplemental Figure 31:** Example case of a normal predicted ECG by DELTAnet. The ECG shows sinus tachycardia, signs of bi-atrial enlargement, left ventricular hypertrophy, and flat repolarization in I and aVL (comparable to previous ECGs). Patient was known with systemic lupus erythematosus (SLE) disease and a cardiologist was consulted because of chest pain complaints and to evaluate whether myocarditis would be a possible diagnosis. Conclusion: complaints not suggestive for myocarditis; at this moment no signs for cardiac involvement. However, cardiac follow-up was recommended (ECG/echo) to rule-out cardiac involvement and medication was changed to optimize hypertension treatment.


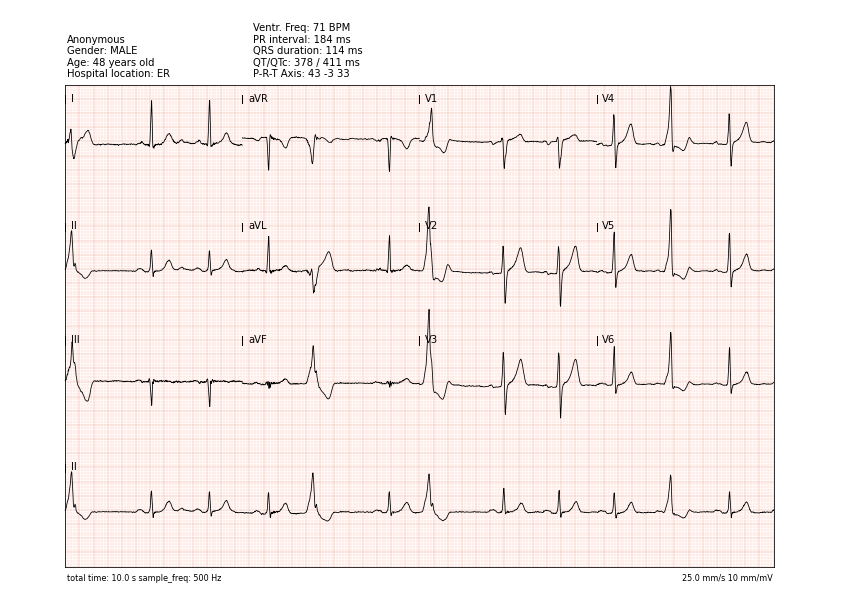
 **Supplemental Figure 32:** Example case of a normal predicted ECG by DELTAnet. The ECG shows normal sinus rhythm with ventricular ectopic beats. The cardiologist was consulted because of chest pain complaints. The cardiologist confirmed a diagnosis of symptomatic ventricular ectopic beats (which the patient was already known with) and recommended a medication change. Patient was already scheduled for follow-up at a cardiology clinic.


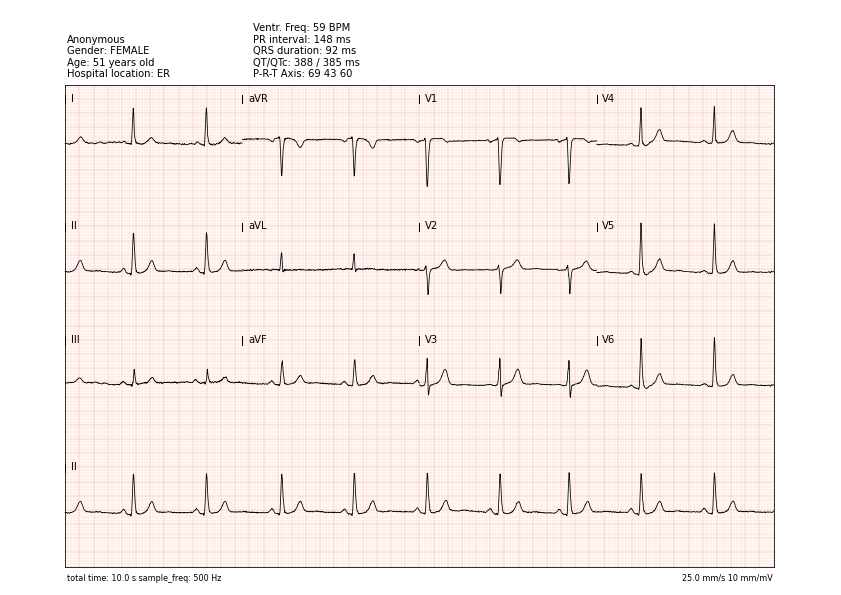
 **Supplemental Figure 33:** Example case of a normal predicted ECG by DELTAnet. The ECG shows normal sinus rhythm. Patient presented with periods of ventricular ectopic beats (not visible at moment of ECG), for which a cardiologist was consulted. The cardiologist changed medication and recommended follow-up at a cardiology clinic.


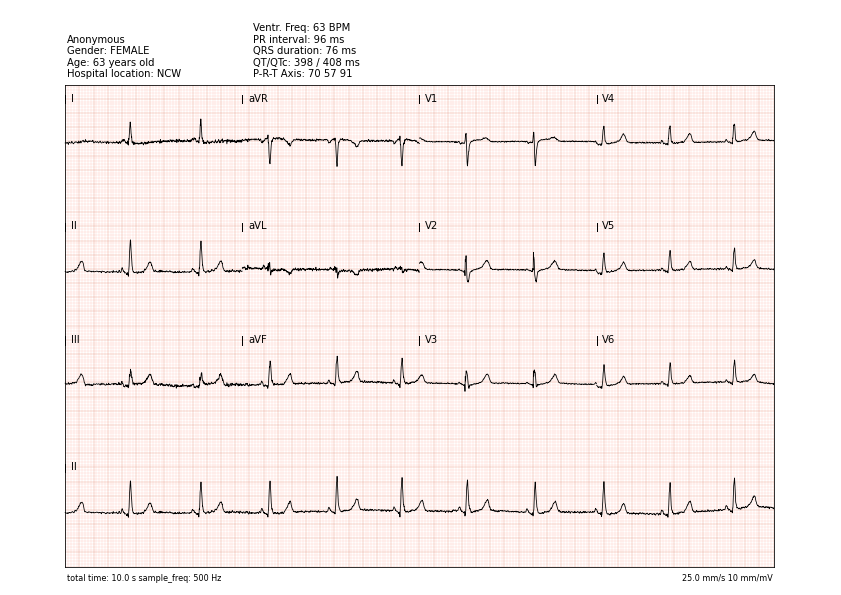
 **Supplemental Figure 34:** Example case of a normal predicted ECG by DELTAnet. The ECG shows normal sinus rhythm. The cardiologist was consulted because of ICD warnings. The patient was admitted because of kidney failure, probably elicited by heart failure (at moment of ECG patient was at a non-cardiology ward). Medication was changed and cardiac follow-up was recommended.


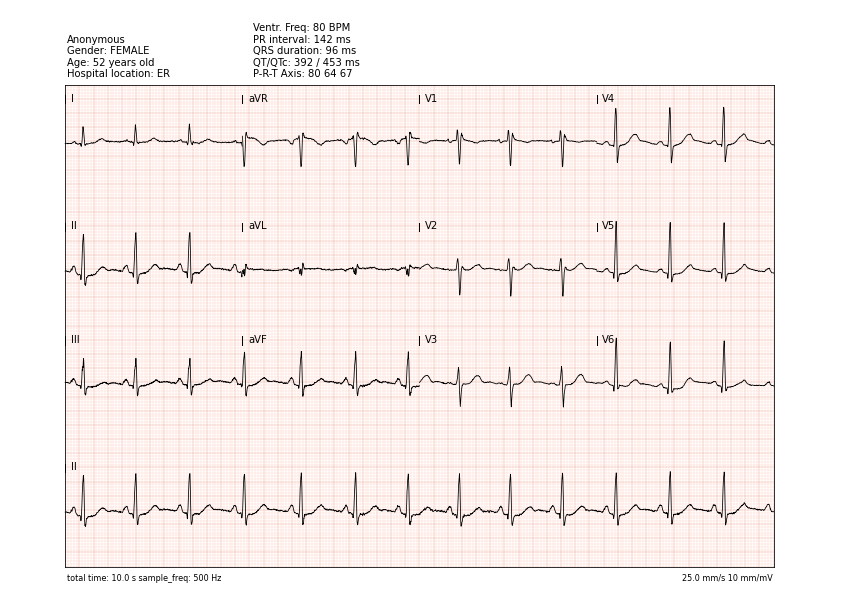
 **Supplemental Figure 35:** Example case of a normal predicted ECG by DELTAnet. The ECG shows normal sinus rhythm with incomplete right bundle branch block. Cardiologist was consulted, because of chest pain complaints with radiation to the left arm. Patient was already known with microvascular coronary dysfunction, however there were no signs for ACS. Patient was given medication and discharged home.


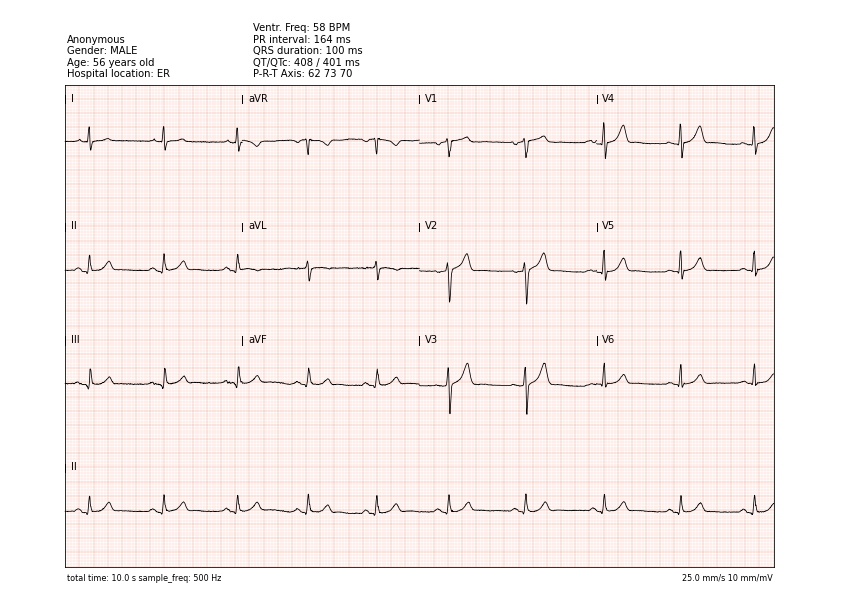
 **Supplemental Figure 36:** Example case of a normal predicted ECG by DELTAnet. The ECG shows sinus rhythm with no abnormalities. The cardiologist was consulted because of chest pain complaints, not primary suspected for ACS. A coronary angiogram was performed to rule out unstable ACS and no significant stenoses were found. Complaints were attributed to be stress-related and patient was discharged home.


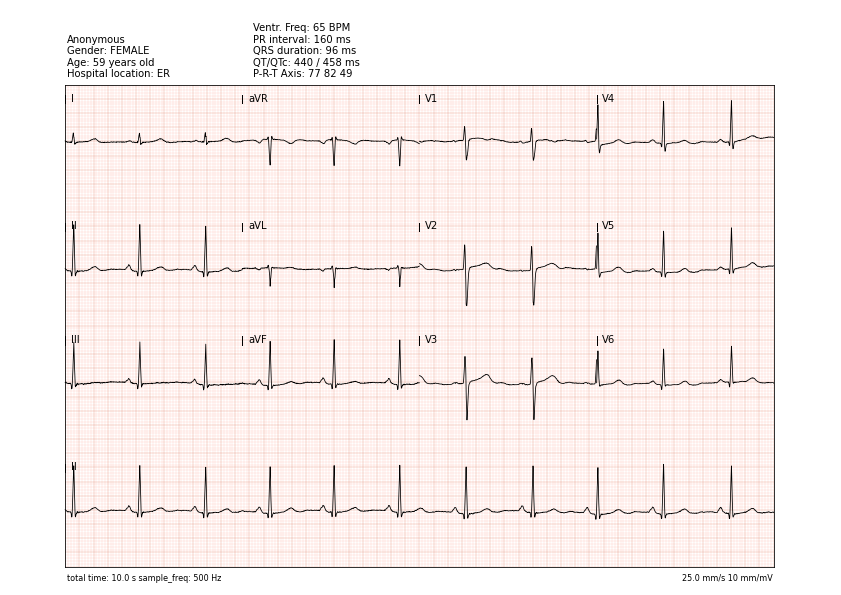
 **Supplemental Figure 37:** Example case of a normal predicted ECG by DELTAnet. The ECG shows sinus rhythm with flat repolarization in III and aVF, no other abnormalities. The patient came in with chest pain complaints (recognized it from earlier myocardial infraction two years ago). Because of chest pain complaints in combination with minimal ECG abnormalities and reduction of complaints when nitroglycerine was given, the patient was suspected for unstable ACS and immediately transferred to the cath lab. Here, a diagnosis of ACS was confirmed and PCI procedure was performed.


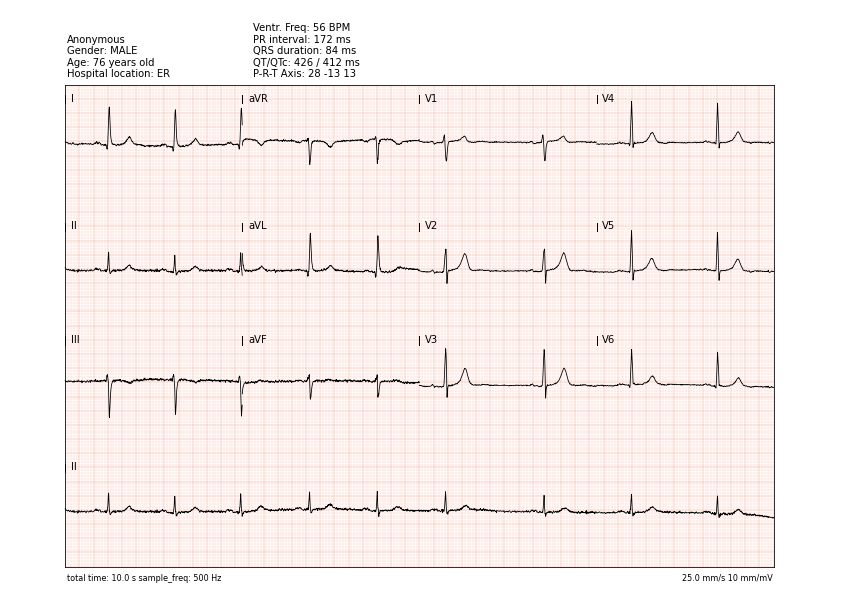
 **Supplemental Figure 38:** Example case of a normal predicted ECG by DELTAnet. The ECG shows normal sinus rhythm with borderline criteria for left ventricular hypertrophy. Cardiologist was consulted because of chest pain complaints. Chest paints complaints were not primary suggestive for ischemia and attributed to be most likely caused by hypertension. 12 days after this ECG, a coronary angiogram was performed and the patient was diagnosed with significant coronary artery disease, for which a CABG procedure was performed.


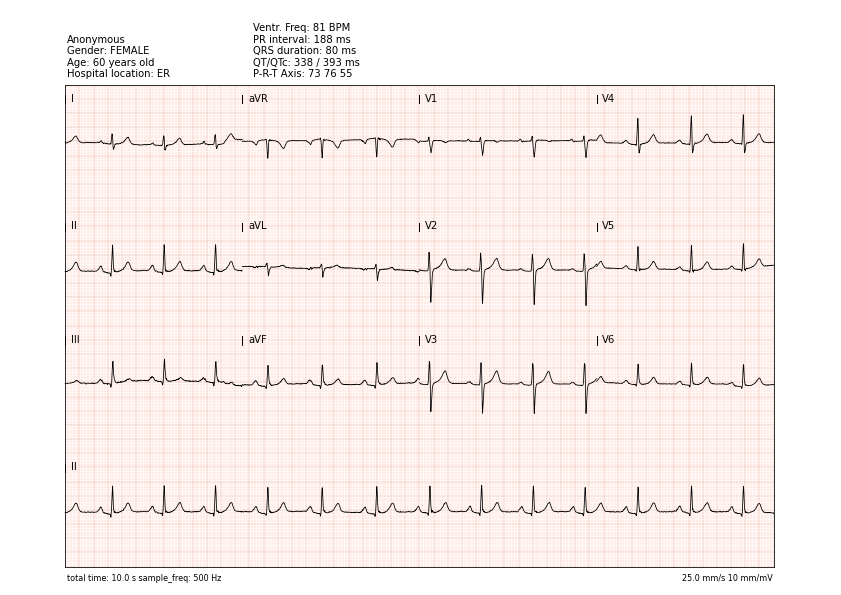
 **Supplemental Figure 39:** Example case of a normal predicted ECG by DELTAnet. The ECG shows normal sinus rhythm, no abnormalities. Cardiologist was consulted because of chest pain complaints. Because of these complaints and elevated troponin, NSTEMI was a possibility diagnosis and a coronary angiogram was performed. However, diagnosis of ACS was not confirmed. No final diagnosis of cardiac disease and patient was discharged home.


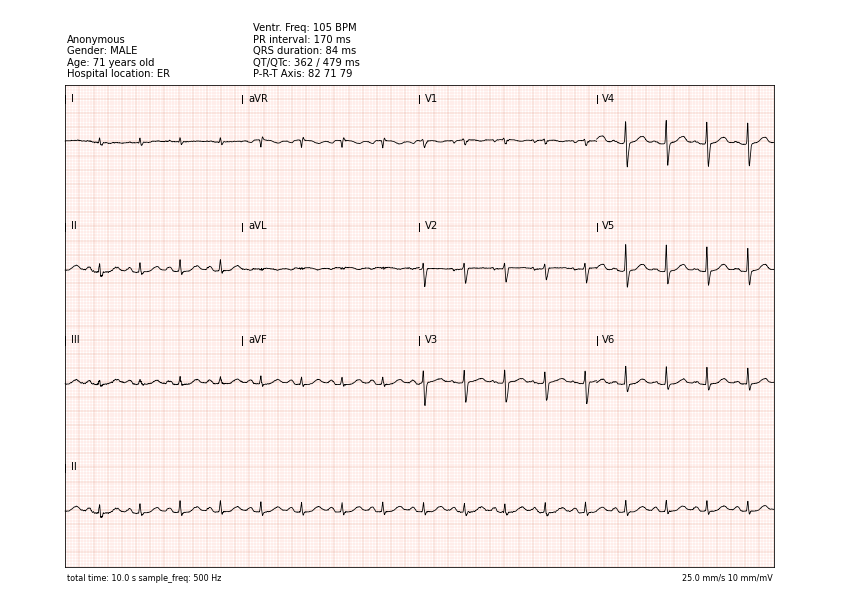
 **Supplemental Figure 40:** Example case of a normal predicted ECG by DELTAnet. The ECG shows tachycardia with low voltages in the extremity leads, no other abnormalities. Patient was referred to the ER because of longer existing dyspnea complaints (cardiologist already in consult before ECG was performed). Pericardial effusion with menacing tamponade was found on a CT thorax. Pericardiocentesis was performed and patient was discharged the next day, follow-up appointment at a cardiology clinic was scheduled.
